# Investigating Tunneling Nanotubes in Cancer Cells: Guidelines for Structural and Functional Studies through Cell Imaging

**DOI:** 10.1155/2020/2701345

**Published:** 2020-04-13

**Authors:** Fatéméh Dubois, Magalie Bénard, Bastien Jean-Jacques, Damien Schapman, Hélène Roberge, Alexis Lebon, Didier Goux, Baptiste Monterroso, Nicolas Elie, Hitoshi Komuro, Céline Bazille, Jérôme Levallet, Emmanuel Bergot, Guénaëlle Levallet, Ludovic Galas

**Affiliations:** ^1^Normandie Université, UNICAEN, CEA, CNRS, ISTCT/CERVOxy Group, F-14000 Caen, France GIP CYCERON; ^2^Service d'Anatomie et Cytologie Pathologiques, CHU de Caen, F-14033 Caen, France; ^3^Normandie University, UNIROUEN, INSERM, PRIMACEN, F-76821 Mont-Saint-Aignan, France; ^4^Normandie Université, UNICAEN, SFR ICORE, Plateau CMABio3, F-14032 Caen, France; ^5^Department of Neuroscience, School of Medicine, Yale University, New Haven, CT 06510, USA; ^6^Service de Pneumologie, CHU de Caen, F-14033 Caen, France

## Abstract

By allowing insured communication between cancer cells themselves and with the neighboring stromal cells, tunneling nanotubes (TNTs) are involved in the multistep process of cancer development from tumorigenesis to the treatment resistance. However, despite their critical role in the biology of cancer, the study of the TNTs has been announced challenging due to not only the absence of a specific biomarker but also the fragile and transitory nature of their structure and the fact that they are hovering freely above the substratum. Here, we proposed to review guidelines to follow for studying the structure and functionality of TNTs in tumoral neuroendocrine cells (PC12) and nontumorigenic human bronchial epithelial cells (HBEC-3, H28). In particular, we reported how crucial is it (i) to consider the culture conditions (culture surface, cell density), (ii) to visualize the formation of TNTs in living cells (mechanisms of formation, 3D representation), and (iii) to identify the cytoskeleton components and the associated elements (categories, origin, tip, and formation/transport) in the TNTs. We also focused on the input of high-resolution cell imaging approaches including Stimulated Emission Depletion (STED) nanoscopy, Transmitted and Scanning Electron Microscopies (TEM and SEM). In addition, we underlined the important role of the organelles in the mechanisms of TNT formation and transfer between the cancer cells. Finally, new biological models for the identification of the TNTs between cancer cells and stromal cells (liquid air interface, *ex vivo*, *in vivo*) and the clinical considerations will also be discussed.

## 1. Introduction

Most of the intercellular communication tools, including the transfer between the gap junctions, extracellular vesicles, and paracrine signals, are well documented. However, the communication through long membrane structures called tunneling nanotubes (TNTs) is still under investigation mainly because of the technical difficulties to study their mechanisms of formation/functioning and the properties that TNTs share.

Through the transfer of organelles, proteins, microRNAs, and ions between cells [1], TNTs are involved in many physiological and pathological processes such as signal transduction, immune response, exchange of micro- and nanoparticles, embryogenesis, cell differentiation and reprogramming, apoptosis, pathogen transfer, and neurodegenerative disease [2]. By allowing “secure” (insured) communication (by cell-cell contact compared to a broadcasted signal) between cells, even distant, of the same type (cancer cells, homo-TNTs) or not (cancer cell-microenvironment cell, hetero-TNTs), TNTs could also be involved in the initiation and growth of cancer then in the dissemination of cancer cells.

Indeed, the cancer development is a multistep process, which results from a complex and continuous interaction between heterogeneous populations of cancer cell per se and noncancer microenvironment cells such as resident fibroblasts and infiltrating immune and inflammatory cells [3]. As emerging components of the intercellular communication, TNTs have been observed between primary cancer cell lines (derived from ovarian, breast, pancreatic, or colon cancer) [4] and in different solid resected tumors from patients [5, 6]. In this regard, TNTs have been increasingly recognized as qualified signals carriers, which can be involved in tumorigenic microenvironment setup. For instance, it has been demonstrated recently that TNTs spread the K-RAS mutation oncogenic message between colon cancer cells, contributing to the tumor heterogeneity and probably to the acquisition of an invasive phenotype of these recipient cells [7]. In addition, several teams describe that TNTs also allow tumor cells to reprogram the healthy neighboring cells to make them more conducive to the formation of a tumor niche. Interestingly, not only a hostile environment (inflammation, hypoxia, and inadequate pH) enhances TNT formation [4, 8–11], but also TNTs could contribute to the escape of the cells from the cell death induced by apoptosis [10, 12–14]. On the other hand, TNTs convey resistance to chemotherapy by exporting the mitochondria [15, 16]. This transfer of mitochondria allows tumor cells to develop one of the hallmarks of tumors, i.e., a metabolic plasticity, in order to acquire migration, proliferation, and resistance properties, particularly to cisplatin and paclitaxel [6, 17].

Many questions about TNTs are still needed to be solved. However, the study of TNTs is technically challenging and requires many precautions to be properly achieved. It means to work in culture conditions preserving the TNTs (TNTs are fragile structures, sensitive to mechanical stress, chemical fixation, light, transfection, trypsinization, cell density, 2D/3D growth culture, cytotoxicity of organelle-labeling reagents, and image acquisition frequency) and to not confuse TNTs with other cytoplasmic expansions (TNTs do not touch the substrate of the culture dish, and extension width must be at more than 1 *μ*m [18]). These conditions vary according to the methodological approach (*in vitro*, *ex vivo*, and *in vivo*) for imaging the TNTs. In this review, we describe the pitfalls which must be avoided for imaging TNTs and we attempt to establish guidelines of the good practice for studying their structure and functionality. These data have been illustrated through different models including tumoral neuroendocrine cells (PC12) and nontumorigenic lung epithelial cells (HBEC-3, H28).

## 2. How Important Are Culture Conditions to Study Connections between Cancer Cells through TNTs?

**Figure 1:**
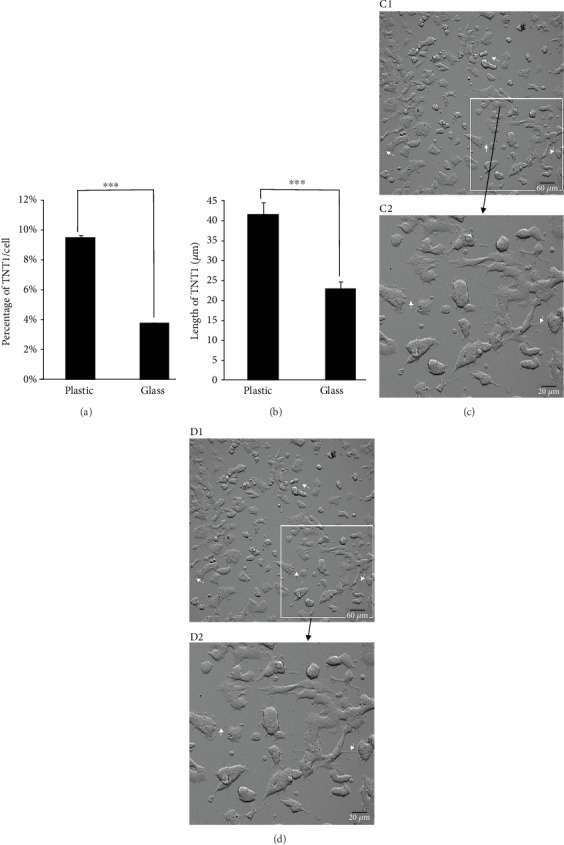
Impact of culture surface on TNT characteristics in PC12 cells. PC12 cells were cultured on a plastic or glass surface for 1 day with similar seeding of 150 000 cells/cm^2^. Histograms showing the impact of plastic or glass surface on the formation on the number (a) and length (b) of TNTs. Experiments were performed 3 times, and at least 200 cells were analyzed for each condition. ^∗∗∗^*P* < 0.001, glass vs. plastic. Images were acquired on a plastic (c) or glass (d) surface with an automated boxed microscope (Celldiscoverer 7, Zeiss) with a 20x dry objective (zoom 0.5 for C1 and D1 or zoom 2 for C2 and D2) through a novel contrasting technique so-called adaptive phase gradient contrast (PGC, Zeiss). A high number of TNTs are detected when PC12 cells are cultured on a plastic surface. In the plastic culture conditions, TNTs are also wider and present more numerous bulging portion (arrows).

Most studies dealing so far with intercellular communication of cancer cells through TNT were performed *in vitro* [2]. Therefore, the determination of culture conditions including surface and cell density, favorable to the formation of homo- and/or hetero-TNTs, is an important milestone in this new cell-to-cell communication process. Here, we report that, in normal culture conditions, plastic and glass surfaces offer properties for TNT formation in neuroendocrine tumoral PC12 cells. However, TNTs are longer and more numerous when PC12 cells are cultured on a plastic versus glass surface (Figure 1). In contrast, the presence or absence of the poly-L-lysine does not influence the number and characteristics of TNT whatever the surface is (not shown). Variation of TNT number could be linked to differential performance of the culture surface for adhesion and migratory processes.

The choice of cell culture products (flask, microplate, and dish) and surfaces is also driven by the type of microscope stand (upright, inverted), the type of microscopy (wide-field, confocal, STED, and SEM) used for the experiments, the characteristics of the objectives (dry, oil, or water immersion), and consequently the expected spatial and temporal resolutions [19]. Microplates or dishes with plastic surface are generally preferred for long time-lapses through bright field, phase contrast, or wide-field fluorescence approaches, i.e., automated boxed microscopes. In contrast, advanced light microscopy (confocal and STED microscopies) requires grade 1.5 (0.17 mm) coverglass in cell cultivation systems (POC chambers, MatTek) for fixed and living cell studies. For scanning and transmitted electron microscopy, all steps of sample preparation are performed with cells attached on coverslips [11].

The main issue for TNT studies is the major impact of cancer cell density due to initial cell seeding and days of culture before observations. Cell density determines the distance and proximity between cells and consequently influences the probability for cells not only to form TNTs but also to form different categories of TNT [20]. As discussed in the next paragraph, cell-to-cell connections through TNTs are a very dynamic process and the proportion of different categories or “intermediate states” of TNT may rapidly differ depending on cell density. As previously described [20], the proportion of TNT1 and TNT2 varied according to PC12 cell density, with a majority of TNT1 one day after plating when cell density was low and a higher number of TNT2 at later times when cell density increased (Supplementary Figure 1). Another point requiring vigilance is the mandatory days of culture after passages or EDTA/trypsin treatments for full membrane recovering important for the formation of TNTs. Similarly, the possible impact of transfection (lipofection or electroporation) on TNT formation should be carefully considered. In addition, gentle fixation (PFA 4%, 2-5 min) for light microscopy and glutaraldehyde (2.5%, 1 h at 4°C) for SEM is recommended due to the fragility of TNTs.

**Figure 2:**
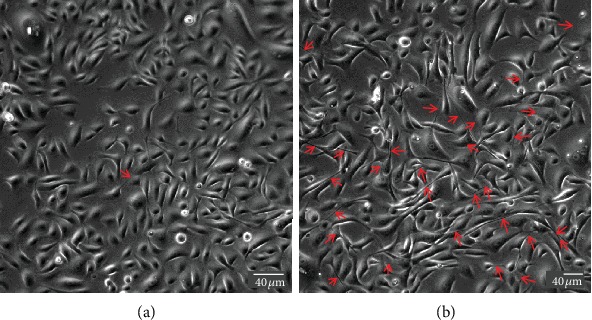
Effect of RASSF1A inhibition on the formation of TNTs in HBEC-3 cells. HBEC-3 cells were cultured on a glass surface, and images were acquired through phase contrast microscopy (DMI 6000 video microscopy, Leica Microsystems). siRNA for RASSF1A [11] treatment was performed at 30% confluence using Lipofectamine RNAiMAX (Invitrogen). TNTs were visualized 2 days after transfection (red arrows).

As cancer cell models, neuroendocrine (PC12), mammary epithelial (MCF-7), pleural mesothelial (H28 and H2052), and bronchial epithelial (A549, H441, H23, and H1650) cell lines exhibit spontaneous formation of TNTs when cultured *in vitro* [11, 20–22]. Of particular interest, human bronchial epithelial HBEC-3 cells, which are nontumorigenic, rarely form TNTs when cultured *in vitro* [11]. However, inhibition through siRNA of RASSF1A, a gene involved in the regulation of cytoskeleton preventing the tumorigenic process (for review [23]), results in a strong increase in the number of TNTs (Figure 2). This suggests that both types of cell lines, forming spontaneously numerous or rare TNTs, respectively, are key cell models to determine pivotal genes involved in their mechanism of formation [11].

Altogether, these data indicate that several parameters including surface of culture, cell density, and transfection steps should be considered to avoid pitfalls when studying TNTs in cultured cells.

## 3. How Important Is It to Visualize the Formation of TNTs in Living Cancer Cells?

**Figure 3:**
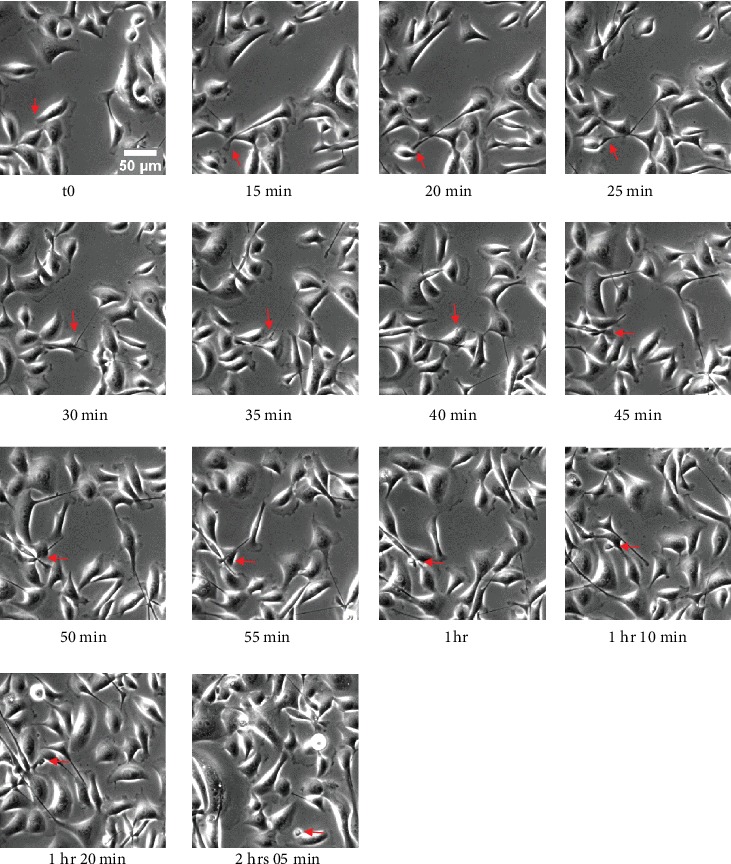
Formation of TNT through directed filopodia-like protrusion in HBEC-3 cells. HBEC-3 cells were cultured on a glass surface, and time-lapse was acquired through phase contrast microscopy (dry 20x, DMI 6000 video microscopy, Leica Microsystems). Red arrows indicate sequential steps of TNT formation. Scale bar, 20 *μ*m.

**Figure 4:**
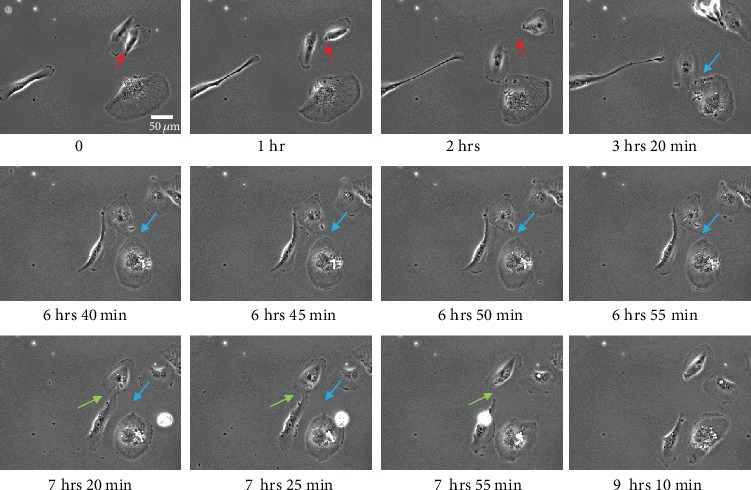
Formation of TNT through cell dislodgement in H28 cells. H28 cells were cultured on a glass surface, and time-lapse was acquired through phase contrast microscopy (dry 20x, DMI 6000 video microscopy, Leica Microsystems). Colored arrows (red, blue, and green) indicate the formation of 3 different TNTs. Scale bar, 20 *μ*m.

**Figure 5:**
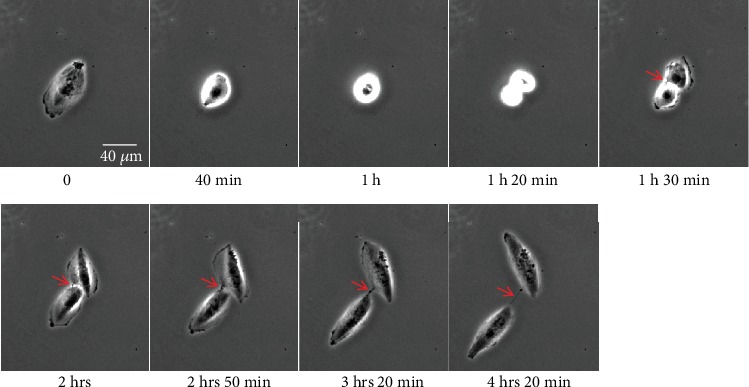
Cell division and cytodieresis in H28 cells. H28 cells were cultured on a glass surface, and time-lapse was acquired through phase contrast microscopy (dry 20x, DMI 6000 video microscopy, Leica Microsystems). Red arrow indicates the successive steps of cytodieresis residue formation.

**Figure 6:**
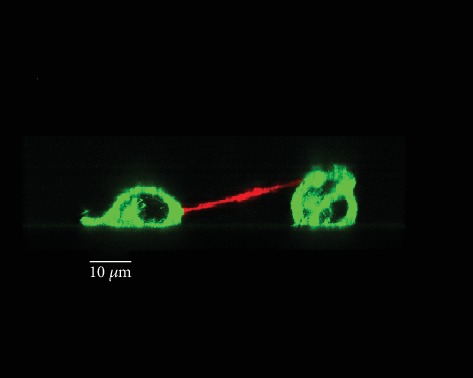
3D representation of TNT connecting PC12 cells through confocal microscopy. Plasma membrane of living PC12 cells was labeled with Alexa-coupled wheat germ agglutinin (WGA), and Z-stack acquisitions (*x*, *y*, *z*) were performed through confocal microscopy (TCS SP5 X, Leica Microsystems). 3D representation of oblique TNT was obtained through Imaris (Bitplane).

TNTs belong to the family of membrane protrusions (intercellular bridges, filopodia, and cytonemes) that connect cells to each other [2, 24]. Due to cell density and rapid membrane dynamics of protrusions, it is sometimes confusing to clearly identify TNTs between cultured cells. Here, we proposed few guidelines to be aware that (i) all cell-to-cell protrusions are not TNTs, (ii) two main mechanisms of TNT formation are generally described, and (iii) categories and morphological variations of TNTs do exist as well as “intermediate states” that could be observed during dynamics of TNT formation or failure. In this context, extensive knowledge of cultured cells through bright-field, phase contrast microscopies (3D: *x*, *y*, *z*, light source with low energy) is of particular interest for visualization of cell movements and cell contacts. For this, types of microscope objective and phase contrast devices as well as high-resolution cameras (sCMOS or CCD) are also crucial to obtain efficient contrast. As shown in Supplementary Figure 2, ×20 objective combined with an additional digital zoom offered better contrasted image (A2, phase ring 2) compared to ×63 objective (A1, phase ring 3) and TNTs are consequently highlighted. Brightfield (B) and adaptive phase gradient contrast (C) technologies provided similar contrast whatever the objective but at the expense of image quality when digital zoom was applied (B2, C2) (Supplementary Figure 2). Long time-lapses through phase contrast microscopy were particularly appropriate to identify (i) the formation of TNT via directed filopodia-like protrusions (Figure 3+movie 1 supp) or cell dislodgement (Figure 4+movie 2 supp) and (ii) residue of cytodieresis (Figure 5+movie 3 supp). As a matter of fact, a residue of cytodieresis could be unfortunately named TNT and a midbody considered a TNT bulging portion if the observation is only performed at “*t* = 4 hrs 20min” (Figure 5). In the case of formation through directed filopodia-like protrusion mode, time-lapses also facilitate the determination of “donor cell” and “acceptor cell” as well as the movement direction of the TNT-bulging portion (Supplementary Figure 3) [20]. In addition to the brightfield and phase contrast imaging, holotomographic microscopy may bring new insights in the future by reducing light exposure. Fluorescence microscopy offers also many possibilities to visualize and analyze TNTs. Fluorescent labeling of plasma membrane with Alexa-coupled wheat germ agglutinin (WGA) (Supplementary Figure 4) combined to Z-stack acquisitions (*x*, *y*, *z*) through confocal microscopy and image analysis allowed a 3D representation of intercellular communication. This rapid approach is the only one providing the evidence that a protrusion is really hovering freely above a substratum [15, 22]. Most TNTs are parallel to the surface of culture, but some are more or less angular depending on cell density and step of formation (Figure 6). A number of morphological criteria including length, diameter, origin, and anchoring aspects have to be also considered to assure a TNT identification. Since intercellular connection is a dynamic process, these characteristics can be rapidly modified during observations. Consequently, we decided to classify TNTs in two categories; i.e., type 1 TNT (TNT1) and type 2 TNT (TNT2). In PC12 and HBEC-3 cells, TNT1 formed a network within groups of cells or joined isolated cells while TNT2 only connected closely adjacent cells [11, 20]. With a length of several tens of *μ*m (up to 300 *μ*m) and a diameter which comprised between 100 and 650 n m, TNT1 are more numerous than TNT2 (max 20 *μ*m long; diameter between 60 and 200 nm) when cells are cultured at a low- to middensity. Identification of two categories of TNTs is intellectually comfortable, but we are also mindful of the number of intermediate states of TNTs due to cell movements, cell proximity, and mechanisms of formation and failure. Again, in a very simple manner, most TNT1 should be considered to be formed through the directed filopodia-like protrusion mode while most TNT2 through the cell dislodgement mode. However, additional mechanism of TNT2 formation by gradual rapprochement of protrusions from both cells may also exist and, depending on cell type, several mechanisms of TNT1 formation are described [25].

Due to cell density, intensity of fluorescent signal, cellular dynamics, and absence of a specific molecular marker, automatic detection of TNTs is rather difficult. Thanks to image processing through ImageJ (NIH) and Huygens professional 18.10 software (SVI, Hilversum), raw images were first deconvoluted and optimized [20]. Then, 3D rendering with Imaris 9.0.2 software (Bitplane) offered the possibility to determine semiautomatically the number and the characteristics of TNTs including their length and width in order to classify TNTs in different categories (Supplementary Figure 5).

Altogether, these data indicate that complementary cell imaging approaches are necessary for correct identification of TNTs including categories, intermediate states, and dynamics of formation. Although phase contrast and confocal microscopies are very useful, these techniques have also spatial limitations to fully resolve the characteristics of TNT. We will see later in this review (Section 5), how STED nanoscopy and transmitted and scanning electron microscopies can break through the barriers of lateral resolution and consequently optimize TNT detection. The details of various imaging methods as well as their comparative advantages and drawbacks are summarized in Table 1.

## 4. How Crucial Is It to Identify Cytoskeleton Components in TNT between Cancer Cells?

Thanks to morphological description and cell dynamics, we have proposed two main categories of TNTs in cancer cells. However, for full characterization, identification of cytoskeleton components and associated elements [2] is required to obtain a comprehensive structural and functional analysis of TNTs.

Actin labeling with Alexa-coupled phalloidin, a toxin that binds to filamentous actin (F-actin), revealed in fixed PC12 and HBEC-3 cells the presence of the protein in both categories of TNTs [11, 20]. Actin was detected as a peripheral thin layer underlying the membrane all along TNT1 delimiting consequently a clear channel between cancer cells [11, 20]. As a crucial cytoskeleton component, actin structured the triangular/trumpet origin of TNT1 as well as the tip presenting a bud-shaped connection or an open-ended tip on acceptor cell [11, 20]. Actin was also observed in TNT2 as a single filament linking two closed cells, but there was no cytosolic channel distinguishable so far through advanced light microscopy (Supplementary Figure 6) [20]. Additionally, TNT1 trumpet-shape origin in H28 and TNT1-bulging portion in HBEC-3 may present actin with short-pick aspect (Supplementary Figure 7). Cofilin, an actin-binding protein, was also detected in TNT1 and TNT2 of HBEC cells (Supplementary Figure 8) [11] suggesting a role in the modulation of actomyosin assembly [26, 27].

**Figure 7:**
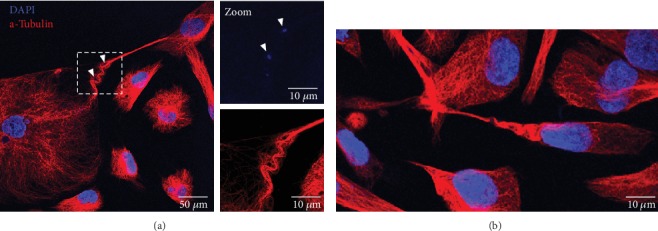
“Accordion-like shape” of tubulin in TNT1 of HBEC-3 cells (a) and H28 cells (b). HBEC-3 and H28 cells were fixed with PFA 4%; then tubulin (red) was stained through immunodetection (Alexa-546). DNA was labeled with DAPI (blue). Image acquisition was captured with high-throughput confocal microscopy (FluoView FV1000, Olympus™).

In contrast to actin, tubulin revealed by an immunocytochemical procedure was only present in TNT1 of PC12 and HBEC-3 cells [11, 20]. A dense network of microtubules delimited both the trumpet-shape origin on donor cell and bud-shaped or open-ended tip on acceptor cell side [20]. A microtubules network was not only restricted to TNT1 by itself but is also described as a continuous network from a donor cell to an acceptor cell suggesting a real tubulin-dependent exchange between cancer cells. In addition, an unusual tubulin structure with an accordion-like shape, never reported elsewhere, was observed in epithelial tumoral H28 cells anchoring TNT1 (Figure 7). This accordion-shape structure might be associated with micronuclei or DNA trail (Figure 7). Another unusual helical organization of microtubules enwrapping intermediate filaments found in TNTs has been observed in cancer urothelial cells [28]. Less “popular” in TNT studies, intermediate filaments are rarely considered [11, 28]. Here, we showed in HBEC-3 cells that vimentin is detected in the cell body of the donor HBEC cell and all along TNT1 including the tip within the acceptor cell (Supplementary Figure 9). Recently, Resnik et al. [29] proposed a triple labelling protocol for actin filaments, intermediate filaments, and microtubules to obtain a full cytoskeleton pattern in cancer urothelial cells.

To monitor actin and tubulin dynamics in living cells, a new generation of probes, i.e., Silicon Rhodamine (SiR), is now available. As an example, SiR actin and tubulin labelled TNTs in living PC12 cells with similar features obtained through immunocytochemical procedures including delimitation of the cytosolic tunnel observed in TNT1 (arrow) (Supplementary Figure 10).

**Figure 8:**
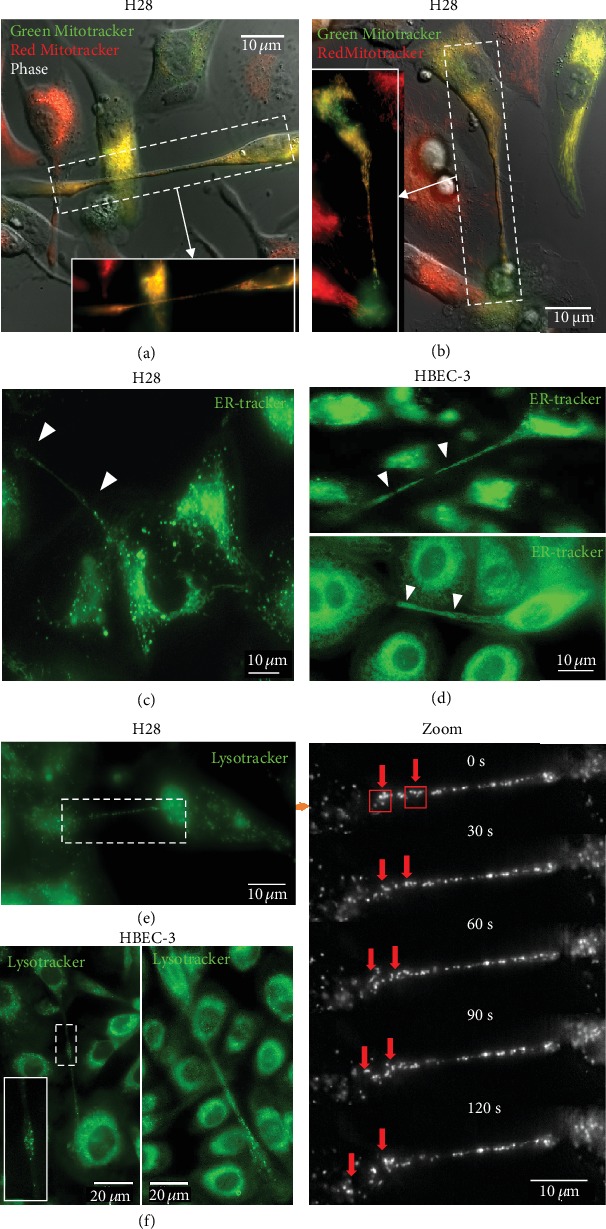
TNTs allow HBEC-3 or H28 cells to exchange organelles. The exchange of organelles was tracked in live HBEC-3 and H28 cells using time-lapse fluorescence video microscopy **(**inverted microscope, Leica DMi8). (a, b) Mitochondria labeled with Mitotrackers are observed inside TNT1s (100x). An example of the mesothelial line H28. Labeling of endoplasmic reticulum with ER-trackers in H28 (c) or HBEC-3 cells (d). Lysosome vesicles in H28 (e) and HBEC-3 (f) cell lines were labelled with Lysotracker and followed by time-lapse performed on an inverted microscope at 100x. (1 image every 30 seconds). The red arrows indicate the passage of the Lysotracker in TNTs to the cell. The Lysotracker and the ER-tracker designated by the white arrowheads are observed within TNTs.

Actin and its associated proteins (myosin, Ral-A, RalGPS2, filamin M-Sec, and Cdc42) as well as vimentin play an important role in the formation of TNTs in various cancer cell models including PC12 cells [30], Hela cells [31, 32], urothelial carcinoma-derived 5637 cancer cells [33], HBEC-3 cells (nontumorigenic) and A549 cells [11], and human osteosarcoma cell line (U2OS) [34]. In bronchial and pleural cell models, cytoskeleton components are controlled through the GEH-H1/Rab11 pathway by RASSF1A which contributes to TNT formation [11]. In contrast, the contribution of tubulin in the mechanisms of TNT formation may be cell type specific since blockage of tubulin did not affect the TNT formation in HBEC-3 cells [11] while stressed PC12 cells form tubulin-dependent TNTs [10]. In cancer cells, the role of TNT microtubules is generally, but not exclusively, associated with the transfer of mitochondria, endoplasmic reticulum, and lysosome vesicles (Figure 8) [2, 17, 25, 35]. While the transport is demonstrated within TNT1 that present a clear cytosolic channel, the existence of cargo-TNT2 is less obvious [20].

Altogether, these data indicate that identification of cytoskeleton components is essential to determine the categories, the mechanisms of formation, and transport/transfer potential of TNTs.

## 5. What Is the Input of STED, TEM, and SEM Approaches for the Characterization of TNTs in Cancer Cells?

**Figure 9:**
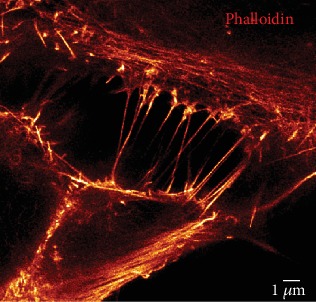
TNT2 imaging in HBEC-3 cells trough STED nanoscopy. HBEC-3 cells were fixed with PFA 4%, and actin labeling was performed with Alexa-488 phalloidin. Image acquisition was obtained through gCW STED nanoscopy (TCS SP5 X, Leica Microsystems). Scale bar, 1 *μ*m.

Depending on fluorophore and numerical aperture of the objective, the lateral resolution reachable through confocal microscopy is comprised between 200 and 250 nm [19]. Due to the nanostructure of TNT, the performance of confocal microscopy could therefore be insufficient to “dissect” tiny TNT elements such as origin/birthplace, attachments, cytosolic tunnel, and cytoskeleton networks. More recently, new developments in fluorescence microscopies including nanoscopy STED combined with image processing (deconvolution) offer a huge three- to fivefold increase in lateral resolution [19]. As an example, microtubule width in PC12 cells differs from 225 to 76 nm depending on cell imaging approaches (Supplementary Figure 11). By using gated CW STED nanoscopy, TNT1 and TNT2 have been characterized in more detail in fixed and living PC12 and HBEC-3 cells [11, 20]. In both cell types, TNT1 exhibited close-ended to open-ended tips suggesting intermediate state during formation and direct cytoplasm connection, respectively [11, 20]. With STED nanoscopy, TNT2 did not show any clear cytosolic tunnel but branched or bulbous attachments on connected HBEC-3 cells were observed (Figure 9). Very recently, live cell STED nanoscopy revealed the involvement of membrane Hsp70 in the formation of TNTs in U87, GL261 glioblastoma, and 4T1 mammary carcinoma cells [36]. Related to depletion laser power, STED nanoscopy may be deleterious for transport/transfer studies through time-lapses [20]. Newly release, less powerful depletion wavelength (775 nm), and ×93 objective (NA = 1.3) will preserve living cells and TNTs making possible multilabelling STED experiments in living cells.

**Figure 10:**
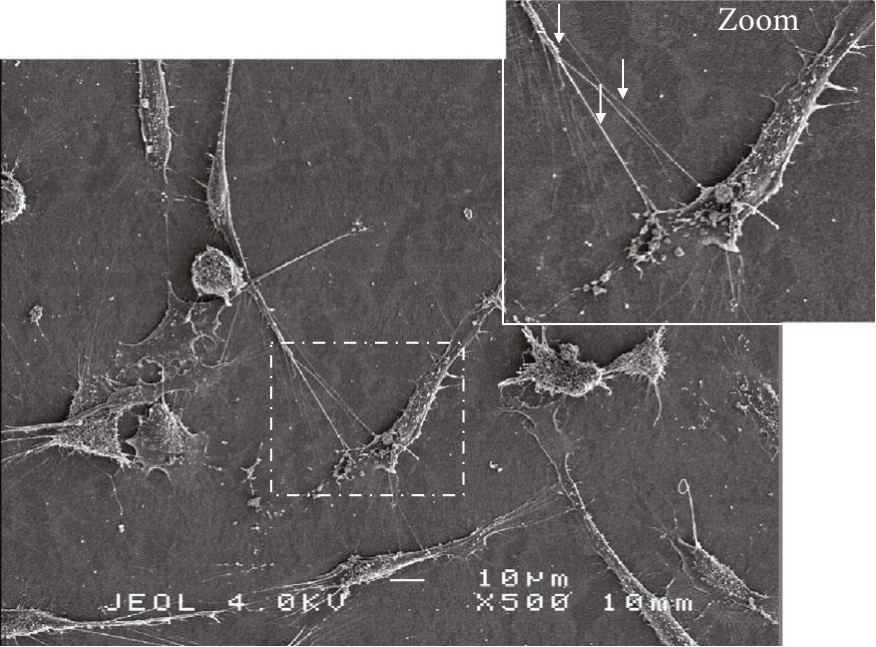
Characterization of TNTs through scanning electron microscopy in HBEC-3 cells. Representative image of TNT between HBEC-3 cells using scanning electron microscopy following fixation with 2.5% glutaraldehyde and dehydration as previously described [11]. The red arrows designate TNTs and their branch lines. Scale bar, 10 *μ*m.

On fixed cells, electron microscopies bring additional details with a lateral resolution less than 1 nm. In particular, Rustom et al. [21] revealed cytosolic channel for short TNTs, probably TNT2, through transmitted electron microscopy. With a specific sample preparation, scanning electron microscopy (SEM) offers the possibility of 3D rendering revealing the position of TNTs above the coverglass. In addition, SEM underlines in more detail membrane aspects of TNTs, the coexistence of single and branched TNTs, the anchoring to the target cell, and more generally the complexity of protrusions and processes in a cancer cell culture (Figure 10). Long single TNTs may in fact result from the association of several TNTs or possibly with multiple insertion points in the cell membrane [4, 37]. Cryoelectron tomography recently developed by Sartori-Rupp et al. [38] in neuronal cells (mouse catecholaminergic CAD cells and human neuroblastoma SH-SY5Y) offers new possibilities to obtain more details and to compare categories and intermediate states of TNTs. In these cells, TNTs are composed of a bundle of open-ended individual tunneling nanotubes (iTNTs) [38].

## 6. How Important Is It to Consider Organelles in the Mechanisms of TNT Formation and Transfer between Cancer Cells?

TNTs facilitate the direct transfer of various cargos, including cytosolic molecules and organelles between both close and distant cells [11, 18]. It has been suggested that organelle transfer through TNTs influences the tumor development, progression, metastasis, and therapeutic response [5, 15, 39, 40]. In this regard, fluorescence microscopy and high-resolution time-lapse imaging aided in understanding the dynamics of the movement in quantitative and qualitative terms. A wide array of fluorescent dyes that target specific organelles is the essential part of the toolbox for direct observation of the TNT activity. These probes not only are highly selective and noninvasive but also offer simple operation and high-resolution signals. For example, Green and/or Red Mitotrackers (Molecular Probes) are the most popular commercially available fluorescent probes used for live cell staining of mitochondria (Figures 8(a) and 8(b)). To determine the transport of mitochondria, treated cells should be divided into two groups after trypsinization and be labeled separately by either Green or Red Mitotracker dyes for 30 min. After extensive washing with PBS, two populations are seeded together in the same culture dish and are subjected to the real-time imaging after one hour of incubation at 37°C. The yellow-colored cells indicate colocalization of the two dyes within the same cell. The transfer of endoplasmic reticulum or lysosome between the cells is performed by adding ER-tracker or Lysotracker, respectively, into normal growth media and then analyzed immediately by real-time acquisition (Figures 8(d)–8(f)). However, long-term and continuous real-time visualization of the organelles probes has been challenging due to the low photostability and dramatic decrease of the signal during imaging. Therefore, the experiments using fluorescent dyes need to be accomplished within 30-45 minutes of acquisition. Moreover, it should be kept in mind that the use of fluorescent molecule can alter organelle morphology and behavior. For example, mitochondria usually fragment in response to the foreign chemical [41]. Furthermore, precautions have to be taken to ensure the correct use of imaging parameters and choice of fluorophore to avoid overlapping of the excitation and emission wavelength.

## 7. Towards New Biological Models to Identify TNTs between Cancer Cells

**Figure 11:**
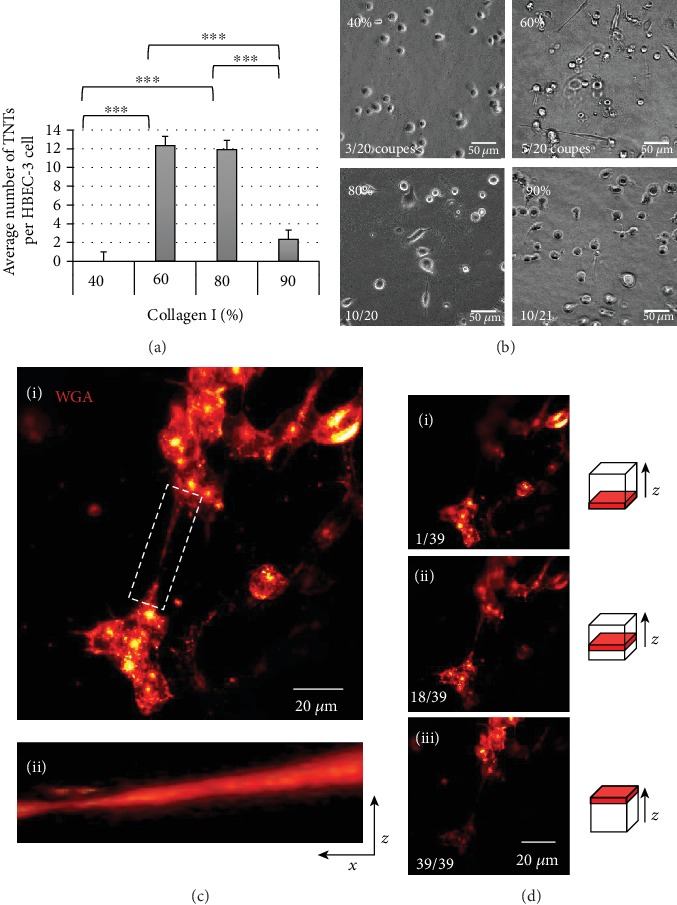
Evolution of the TNT formation according to the concentration of collagen. (a) Quantification and (b) representatives' images of the average number of TNT formation according to the different concentrations of the collagen I: (i) 40%, (ii) 60%, (iii) 80%, and (iv) 90%, performed on an inverted microscope (Leica DMi8) at 40x, cut in the middle of the stack. Data are represented as the mean ± SEM from three individual experiments. For each experiment, 100 cells were considered on an average. *P* values were obtained using Student's unpaired *t*-test. ^∗∗∗^*P* < 0.001. (c) (i) Representative images of the 3D reconstitution (max projection) of the lectin-labeled HBEC-3 cells (wheat germ agglutinin- (WGA-) 633) cultured in the middle of the collagen layers. (ii) The ImageJ software program (image/stacks/3D project) was used for 3D reconstruction. (d) Representative images of the single slices of Z-stacks on (i) top, (ii) middle, and (iii) bottom of collagen layers. Acquisition with a confocal microscope, Olympus FV1000, objective 40x. Z-stacks were generated by taking optical sections at 0.5 *μ*m intervals.

Consideration of a more physiologically relevant microenvironment for the evaluation of the TNTs formation is often obtained through *in vitro* assay of cells grown in spheroid/organoid culture [42] and/or *in vivo* rodent carcinogenesis models [43, 44] as well as direct analysis of patient biopsies and surgical samples [4, 5, 45]. However, because of the absence of the space between the cells, microscopic imaging of the formed TNTs in these experimental systems is hindered by different factors such as high background, poor signal, and low resolution of the images. To overcome the problems of the limited accessibility and manipulability and to further confirm TNT formation in a 3D environment, our research group plate the cells between two layers of type I collagen matrices [11] (Figure 11). This method provides the advantage of 3D culture while preserving the distance between individual and separate cells [46].

**Figure 12:**
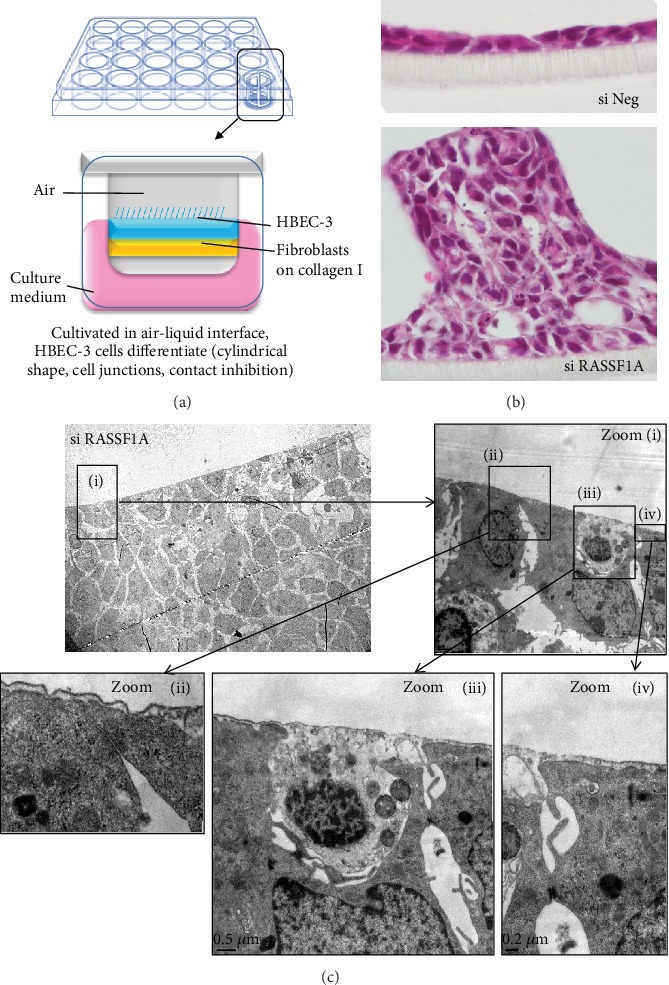
Observation of TNTs in scanning electron microscopy of a culture of HBEC-3 cultivated in an air-liquid interface. In an air-liquid interface whose procedure is illustrated in (a), HBEC-3 cells established a single layer of columnar cells (b), while RASSF1A-depleted HBEC-3 cells exhibited focal features of hyperplasia (b). The observation by scanning electron microscopy of RASSF1A-depleted HBEC-3 cultures ((c), different zooms are shown from (i) to (iv)) reveals the presence of long cellular extensions mainly carried by cells without RASSF1A transparent with electrons, delimiting the cellular clusters.

It has long been criticized that what we know about TNTs was obtained from cells grown in 2D, a legitimate criticism since in the organism; no cell is found in this state. Some critics even argued that what we were observing was actually artifacts related to this mode of cell culture. 3D culture partly responds to these criticisms (Figure 11); it reproduces a three-dimensional matrix closer to what is usually experienced by cells, including tumors. A new approach is also to observe and study these TNTs in a much more physiological context, as for example, in *ex vivo* slice. For epithelial cells which are polarized cells, we can develop culture models which mimic their natural organization in the organism, for example, for human bronchial cells, by carrying out cell cultures at the air-liquid interface [47] (Figure 12(a)).

As we have already reported, thus cultivated HBEC-3 cells which are immortalized epithelial bronchial cells (nontumorigenic) like a bronchial epithelium form only one layer of cells (the inhibition of contact is functional); the cells are cylindrical and establish junctions between them but on the other hand fail to form eyelashes (Figure 12(b) si Neg). When the expression of the scaffolding protein RASSF1A, in charge of microtubule and actin dynamics, is extinguished by means of siRNA, the cells no longer undergo contact inhibition and thus form several strata; their forms are more random from squamous to ovoid, and the cell junctions are less dense (Figure 12(b), si RASSF1A). Unexpectedly, the appearance of the cell mass is very regular/linear or even geometric (cubic). Surprised after this aspect, we observed these strata of cells under the transmission electron microscope and discovered that this mass of cells harbors two types of cells: some are dense with electrons; others are not (Figure 12(c)). The cells transparent with electrons also form long cell extension that are compatible with that of a TNT, which cover the other cells and seem to contain the dark cells and explain why this cell mass appears so “square” and regular on its periphery (Figure 12(c)). Once again, we are limited for acquisitions and these TNTs by the optics currently available. For *ex vivo* imaging, tumor samples can be observed through confocal or possibly through light sheet microscopies with nevertheless limiting depth penetration and spatial resolution. *In vivo* imaging could be performed through multiphoton microscopy to collect fluorescent signal up to 800 *μ*m within the tissue. After necessary surgery for tumor access, anaesthetized animals are placed on a fixed stage upright microscope and ×25/×40 objectives combined to additional zoom are used to image cellular structures. Hopefully, the emerging experimental approaches such as correlative light-electron microscopy will expand our understanding of TNT formation in the complex tissue microenvironment.

Finally, the burning question is can we visualize these TNTs on tumor samples of patients? TNTs have been reported *in vitro*, *in vivo*, and *ex vivo*, particularly in tumor samples from patients with malignant pleural mesothelioma (MPM) and adenocarcinoma, but their identification remains tedious, based mainly on visual identification of morphological and/or functional characteristics (by highlighting an intercellular cargo transfer). The difficulty in their identification lies in the fact (i) that it is a fragile structure, sensitive to light, oxidative stress, and chemical fixation [48] and (ii) that no specific or universal marker has yet been reported, explained by the heterogeneity of these structures, revealed by the growing number of publications on the subject, in which most of the molecular candidates studied were probably more cell-specific than TNT-specific [49]. However, the description of proteins found in several cell types and involved in the formation of TNTs argues in favor of their specificity towards TNTs. Thus, the identification of several proteins, constituting a molecular signature, testifying to the presence of TNTs, appears more appropriate. Among these proteins are (i) the cytoskeleton element (vimentin [11], tubulin, and actin) and consequently the proteins involved in their remodeling (LST1, Filamin, Ral-A GTPase, M-Sec, CDC42, etc.) [50]; (ii) the i-BAR protein, which plays a role in the stretching of the plasma membrane; and (iii) the Akt/PI3K/mTOR signaling pathway members [51], but this alternative of a molecular signature would make the application in routine laboratory complex and expensive.

To resolve this, we can perhaps associate the positivity of immunostaining with its appearance: we knew that vimentin is present in TNTs, in particular in TNT1, carrying immunostaining of vimentin on tumor samples from 10 patients with malignant pleural mesothelioma and look for long cellular extensions. The remaining question is are these TNTs correlated with overall survival or progression-free survival? A future work focusing on trying to identify these TNTs in patients with cancer will have to answer this question by correlating the quantity of TNTs with the survival data of these patients.

## 8. Conclusions and Perspectives

At the end of this review, we can more than ever recognize the complexity to identify with certainty a TNT and not to confuse it with another cytoplasmic expansion, so we recommend the following approaches:

- Monitoring TNT formation and disruption in living cells with low-energy light source and through bright-field or phase contrast microscopy (or similar approach)
- Determining the *z* position of TNT through 3D representation through confocal microscopy
- Determining the “most real” size of TNT through advanced light (STED) and electron microscopies (TEM, SEM, and tomography) and correlative strategies
- Determining the cytoskeleton components in TNT subtypes or intermediate states of TNTs
- Developing appropriate image processing to characterize TNTs (number, length).

We also propose to pay attention to the impact of culture surface (glass, plastic, and coating), cell density (50% is preferred), stresses (trypsinization, transfection, and drug treatment), and chemical fixatives on TNT number and properties. As additional recommendations, we should be also aware of the potential toxic effect of “vital” probes [19] and light sources (lasers, mercury of metal halide lamps) during long time-lapses through fluorescence microscopies. This caution is mandatory when transfer mechanisms (organelles, molecules, pathogens) through TNT are studied. In addition, we suggest to propose to characterize TNTs or TNT-like structures in 3D microenvironments including matrices, spheroids/organoids, biopsies, or surgical samples to consider more physiological or physiopathological conditions.

It is also complex to identify who are the protagonists of the formation and functionality of TNTs, since these cytoplasmic expansions will necessarily include elements of the plasma membrane or from the cytoplasm present everywhere else in the cell. Modulation approaches for the expression of the proteins that are thought to be involved in these TNTs, by silencing (siRNA, shRNA) or, on the contrary, overexpression, can make it possible to see if the amount of these proteins is correlated with the amount of TNTs and to provide strong arguments to suggest their involvement in these structures.

Although the study of TNTs is still in its infancy, the growing number of publications on the subject suggests for this innovative field of study many applications in both cancer therapy and anticancer therapies, infectious diseases, neurodegenerative, or cardiovascular. That is why improving our knowledge on these TNTs is so important; it could even represent a major stake in our fight against pathological processes. TNTs could play a role, for example, in cellular rescue/restoration *via* mitochondrial transfer in the context of ischemic cell damage associated with infarction [52]. The knowledge we have acquired about the properties of these TNTs and their implications for tumorigenic processes offers some perspectives for the new management of cancer patients:

- To inhibit their training and therefore their function: this approach is particularly developed in the fight against infectious diseases and especially HIV. Several molecules, targeting different signaling pathways (in particular mTOR, NF-KB, Rac1, or inhibiting the actin polymerization or the assembly of microtubules), allowing the inhibition of TNTs have been described (everolimus, metformin, cytochalasin D, nocodazole CK-666, ML-141, 6-thio-GTP, latrunculin A, latrunculin B, cytarabine, daunorubicin, BAY-117082, AGA, and octanol) [52]. This approach appears fully applicable to anticancer therapies, limiting the transfer of biological material between cancer cells and/or microenvironment cells. In this sense, a study shows in a cellular model of acute T lymphoblastic leukemia that the inhibition of ICAM-1, an adhesion molecule, decreases the mitochondrial transfer mediated by mesenchymal stem cells *via* TNTs, responsible for an increase of cell death induced by the associated chemotherapy [53]
- To use TNTs as tools for vectorization of drugs between cancer cells: indeed, it has been shown that polymeric nanoparticles, a medical device for the delivery of active principles, could be internalized and transferred from one cell to another *via* the TNTs. This approach, coupling polymeric nanoparticles as a vector for anticancer therapy, would make it possible to homogenize the distribution of the drug to all the cellular components of the tumor, particularly in a hypoxic context [54]
- To use the ability of TNTs to promote cell survival as part of cellular therapies: the latter approach concerns cell therapies or stem cell transplantation, treatment of leukemia, and certain non-Hodgkin's lymphomas, whose main limitation is the senescence acquired by grafted cells limiting their effectiveness over time. One study demonstrated that the culture of these mesenchymal stem cells in three-dimensional structures (spheroids) compared to two-dimensional culture favored the formation of TNTs, the intercellular exchange of cytoplasmic components, and modulated expression of senescence markers and ultimately improve the survival of these transplant cells [55].

## Figures and Tables

**Table 1:** Imaging methods and fluorescent markers for TNT studies: the pros and the cons.

Imaging methods	Fluorescent markers/dyes for TNT studies	Pros for TNT imaging	Cons for TNT imaging
Bright-field/phase-contrast microscopy Inverted stand is mandatory	—	Living cells Absence of exogenous markers/dyes Most cell culture product (flask, microplate, dish) Time-lapse	Spatial resolution No Z-stack

Holotomographic microscopy	—	Living cells Absence of exogenous markers/dyes Dish with coverglass Low phototoxicity Time-lapse	To be determined in the next future

Wide-field fluorescence microscopy Any fluorophore consistent with filters and spectral sensitivity of detectors Inverted stand is mandatory for living cells	(i) Fluorophore-coupled lectin (mostly Alexa-WGA). Living and fixed cells Plasma membrane labeling Intracellular vesicle labeling can also be observed depending on used concentration and time of incubation (ii) Fluorophore-coupled phalloidin for detection of actin in fixed cells (iii) SiR actin for detection of actin in living cells (iv) Antibodies directed against tubulin, vimentin, and cofilin for immunocytochemistry (v) SiR tubulin for detection of tubulin in living cells (vi) Mitotrackers (green, red) for detection of mitochondria in TNTs (mostly living cells) (vii) ER- and Lysotrackers for detection of endoplasmic reticulum and lysosomes in TNTs (mostly living cells) (viii) Any fluorescent protein constructs for the identification of proteins involved in TNT formation or transport along TNTs	Living and fixed cells	Spatial resolution Photobleaching and Phototoxicity might be important. Red and far-red fluorophores are preferred for living cells studies.
Confocal laser scanning microscopy Any fluorophore consistent with lasers, filters, prisms and spectral sensitivity of detectors Inverted stand is mandatory for living cells		Living and fixed cells Spatial and temporal resolution Z-stack	Grade 1.5 coverglass is mandatory Photobleaching and phototoxicity might be important Red and far-red fluorophores are preferred for living cell studies

Stimulated Emission Depletion (STED) nanoscopy Fluorophore consistent with STED approach and depletion lasers (592, 660, and 775 nm)	Any cellular targets (see above) can be considered for STED imaging but fluorophore compatibility must be checked. Please refer to https://www.leica-microsystems.com/science-lab/the-guide-to-sted-sample-preparation/	Fixed cells Possible but more difficult for living cells High spatial resolution	Grade 1.5 coverglass is mandatory Photobleaching and phototoxicity may be important Restricted to fluorophore consistent with STED

Scanning electron microscopy	—	Very high spatial resolution. 3D rendering.	Time-consuming and sample preparation

Transmitted electron microscopy	—	Very high spatial resolution Cryotomography	Time-consuming and sample preparation

## Supplementary Materials

Supplementary 1 Supplementary Figure 1: impact of cell density on TNT categories in PC12 cells. Supplementary Figure 2: adapted transmitted light microscopies for TNT observation. Supplementary Figure 3: formation of TNT1 in PC12 cells. Supplementary Figure 4: TNT1 and TNT2 imaging in living cancer cells through confocal microscopy. Supplementary Figure 5: confocal image processing and analysis for the determination of TNT features. Supplementary Figure 6: localization of actin and tubulin in TNT1 and TNT2 by double-labeling experiments and confocal microscopy. Supplementary Figure7: actin picks at the surface of TNT1 in cancer cells. Supplementary Figure 8: localization of cofilin in TNTs connecting HBEC-3 cells. Supplementary Figure 9: localization of vimentin in TNT1 connecting HBEC-3 cells. Supplementary Figure 10: localization of actin and tubulin in living PC12 cells through SiR tubulin probe. Supplementary Figure 11: improvement of lateral resolution through advanced light microscopy.

Supplementary 2 Supplementary Movie 1: formation of TNT through directed filopodia-like protrusion in HBEC-3 cells.

Supplementary 3 Supplementary Movie 2: formation of TNT through cell dislodgement in H28 cells.

Supplementary 4 Supplementary Movie 3: cell division and cytodieresis in H28 cells.

## References

[1] Dupont M., Souriant S., Lugo-Villarino G., Maridonneau-Parini I., Vérollet C. (2018). Tunneling nanotubes: intimate communication between myeloid cells. *Frontiers in Immunology*.

[2] Sisakhtnezhad S., Khosravi L. (2015). Emerging physiological and pathological implications of tunneling nanotubes formation between cells. *European Journal of Cell Biology*.

[3] Wang M., Zhao J., Zhang L. (2017). Role of tumor microenvironment in tumorigenesis. *Journal of Cancer*.

[4] Lou E., Fujisawa S., Morozov A. (2012). Tunneling nanotubes provide a unique conduit for intercellular transfer of cellular contents in human malignant pleural mesothelioma. *PLoS One*.

[5] Ady J. W., Desir S., Thayanithy V. (2014). Intercellular communication in malignant pleural mesothelioma: properties of tunneling nanotubes. *Frontiers in Physiology*.

[6] Vignais M. L., Caicedo A., Brondello J. M., Jorgensen C. (2017). Cell connections by tunneling nanotubes: effects of mitochondrial trafficking on target cell metabolism, homeostasis, and response to therapy. *Stem Cells International*.

[7] Desir S., Wong P., Turbyville T. (2019). Intercellular transfer of oncogenic KRAS via tunneling nanotubes introduces intracellular mutational heterogeneity in colon cancer cells. *Cancers*.

[8] Wang X., Veruki M. L., Bukoreshtliev N. V., Hartveit E., Gerdes H. H. (2010). Animal cells connected by nanotubes can be electrically coupled through interposed gap-junction channels. *Proceedings of the National Academy of Sciences of the United States of America*.

[9] Kabaso D., Gongadze E., Jorgačevski J. (2011). Exploring the binding dynamics of BAR proteins. *Cellular and Molecular Biology Letters*.

[10] Wang X., Gerdes H. H. (2015). Transfer of mitochondria via tunneling nanotubes rescues apoptotic PC12 cells. *Cell Death and Differentiation*.

[11] Dubois F., Jean-Jacques B., Roberge H. (2018). A role for RASSF1A in tunneling nanotube formation between cells through GEFH1/Rab11 pathway control. *Cell Communication and Signaling*.

[12] de Rooij B., Polak R., Stalpers F., Pieters R., den Boer M. (2017). Tunneling nanotubes facilitate autophagosome transfer in the leukemic niche. *Leukemia*.

[13] Guo R., Davis D., Fang Y. (2018). Intercellular transfer of mitochondria rescues virus-induced cell death but facilitates cell-to-cell spreading of porcine reproductive and respiratory syndrome virus. *Virology*.

[14] Shen J., Zhang J. H., Xiao H. (2018). Mitochondria are transported along microtubules in membrane nanotubes to rescue distressed cardiomyocytes from apoptosis. *Cell Death and Disease*.

[15] Pasquier J., Guerrouahen B. S., al Thawadi H. (2013). Preferential transfer of mitochondria from endothelial to cancer cells through tunneling nanotubes modulates chemoresistance. *Journal of Translational Medicine*.

[16] Valdebenito S., Lou E., Baldoni J., Okafo G., Eugenin E. (2018). The novel roles of connexin channels and tunneling nanotubes in cancer pathogenesis. *International Journal of Molecular Sciences*.

[17] Hekmatshoar Y., Nakhle J., Galloni M., Vignais M. L. (2018). The role of metabolism and tunneling nanotube-mediated intercellular mitochondria exchange in cancer drug resistance. *Biochemistry Journal*.

[18] McCoy-Simandle K., Hanna S. J., Cox D. (2016). Exosomes and nanotubes: control of immune cell communication. *The International Journal of Biochemistry & Cell Biology*.

[19] Galas L., Gallavardin T., Bénard M. (2018). “Probe, Sample, and Instrument (PSI)”: The Hat-Trick for Fluorescence Live Cell Imaging. *Chemosensors*.

[20] Bénard M., Schapman D., Lebon A. (2015). Structural and functional analysis of tunneling nanotubes (TnTs) using gCW STED and gconfocal approaches. *Biology of the Cell*.

[21] Rustom A., Saffrich R., Markovic I., Walther P., Gerdes H. H. (2004). Nanotubular highways for intercellular organelle transport. *Science*.

[22] Pasquier J., Galas L., Boulangé-Lecomte C. (2012). Different modalities of intercellular membrane exchanges mediate cell-to-cell p-glycoprotein transfers in MCF-7 breast cancer cells. *The Journal of Biological Chemistry*.

[23] Dubois F., Bergot E., Zalcman G., Levallet G. (2019). RASSF1A, puppeteer of cellular homeostasis, fights tumorigenesis, and metastasis--an updated review. *Cell Death and Disease*.

[24] Yamashita Y. M., Inaba M., Buszczak M. (2018). Specialized intercellular communications via cytonemes and nanotubes. *Annual Review of Cell and Developmental Biology*.

[25] Antanavičiūtė I., Rysevaitė K., Liutkevičius V. (2014). Long-distance communication between laryngeal carcinoma cells. *PLoS One*.

[26] Arnold T. R., Stephenson R. E., Miller A. L. (2017). Rho GTPases and actomyosin: partners in regulating epithelial cell-cell junction structure and function. *Experimental Cell Research*.

[27] Wiggan O.’. N., Schroder B., Krapf D., Bamburg J. R., DeLuca J. G. (2017). Cofilin regulates nuclear architecture through a myosin-II dependent mechanotransduction module. *Scientific Reports*.

[28] Resnik N., Prezelj T., de Luca G. M. R. (2018). Helical organization of microtubules occurs in a minority of tunneling membrane nanotubes in normal and cancer urothelial cells. *Scientific Reports*.

[29] Resnik N., Erman A., Veranič P., Kreft M. E. (2019). Triple labelling of actin filaments, intermediate filaments and microtubules for broad application in cell biology: uncovering the cytoskeletal composition in tunneling nanotubes. *Histochemistry and Cell Biology*.

[30] Bukoreshtliev N. V., Wang X., Hodneland E., Gurke S., Barroso J. F. V., Gerdes H. H. (2009). Selective block of tunneling nanotube (TNT) formation inhibits intercellular organelle transfer between PC12 cells. *FEBS Letters*.

[31] Hase K., Kimura S., Takatsu H. (2009). M-Sec promotes membrane nanotube formation by interacting with Ral and the exocyst complex. *Nature Cell Biology*.

[32] Schiller C., Diakopoulos K. N., Rohwedder I. (2013). LST1 promotes the assembly of a molecular machinery responsible for tunneling nanotube formation. *Journal of Cell Science*.

[33] D'Aloia A., Berruti G., Costa B. (2018). RalGPS2 is involved in tunneling nanotubes formation in 5637 bladder cancer cells. *Experimental Cell Research*.

[34] Pergu R., Dagar S., Kumar H., Kumar R., Bhattacharya J., Mylavarapu S. V. S. (2019). The chaperone ERp29 is required for tunneling nanotube formation by stabilizing MSec. *The Journal of Biological Chemistry*.

[35] Burt R., Dey A., Aref S. (2019). Activated stromal cells transfer mitochondria to rescue acute lymphoblastic leukemia cells from oxidative stress. *Blood*.

[36] Reindl J., Shevtsov M., Dollinger G., Stangl S., Multhoff G. (2019). Membrane Hsp70-supported cell-to-cell connections via tunneling nanotubes revealed by live-cell STED nanoscopy. *Cell Stress & Chaperones*.

[37] Lou E., Zhai E., Sarkari A. (2018). Cellular and molecular networking within the ecosystem of cancer cell communication via tunneling nanotubes. *Frontiers in Cell and Developmental Biology*.

[38] Sartori-Rupp A., Cordero Cervantes D., Pepe A. (2019). Correlative cryo-electron microscopy reveals the structure of TNTs in neuronal cells. *Nature Communications*.

[39] Desir S., Dickson E. L., Vogel R. I. (2016). Tunneling nanotube formation is stimulated by hypoxia in ovarian cancer cells. *Oncotarget*.

[40] Haimovich G., Ecker C. M., Dunagin M. C. (2017). Intercellular mRNA trafficking via membrane nanotube-like extensions in mammalian cells. *Proceedings of the National Academy of Sciences of the United States of America*.

[41] Mitra K., Lippincott-Schwartz J. (2010). Analysis of mitochondrial dynamics and functions using imaging approaches. *Current Protocols in Cell Biology*.

[42] Zhang J., Whitehead J., Liu Y., Yang Q., Leach J. K., Liu G. Y. (2018). Direct observation of tunneling nanotubes within human mesenchymal stem cell spheroids. *The Journal of Physical Chemistry B*.

[43] Chinnery H. R., Pearlman E., McMenamin P. G. (2008). Cutting edge: membrane nanotubes in vivo: a feature of MHC class II+ cells in the mouse cornea. *The Journal of Immunology*.

[44] Osswald M., Jung E., Sahm F. (2015). Brain tumour cells interconnect to a functional and resistant network. *Nature*.

[45] Rehberg M., Nekolla K., Sellner S. (2016). Intercellular transport of nanomaterials is mediated by membrane nanotubes in vivo. *Small*.

[46] Artym V. V., Matsumoto K. (2010). Imaging cells in Three-Dimensional collagen matrix. *Current Protocols in Cell Biology*.

[47] Dubois F., Keller M., Calvayrac O. (2016). RASSF1A suppresses the invasion and metastatic potential of human non-small cell lung cancer cells by inhibiting YAP activation through the GEF-H1/RhoB pathway. *Cancer Research*.

[48] Panasiuk M., Rychłowski M., Derewońko N., Bieńkowska-Szewczyk K. (2018). Tunneling nanotubes as a novel route of cell-to-cell spread of herpesviruses. *Journal of Virology*.

[49] Lou E., Gholami S., Romin Y. (2017). Imaging tunneling membrane tubes elucidates cell communication in tumors. *Trends Cancer*.

[50] Sun J., Kawakami H., Zech J., Speck C., Stillman B., Li H. (2012). Cdc6-induced conformational changes in ORC bound to origin DNA revealed by cryo-electron microscopy. *Structure*.

[51] Kretschmer A., Zhang F., Somasekharan S. P. (2019). Stress-induced tunneling nanotubes support treatment adaptation in prostate cancer. *Scientific Reports*.

[52] Mittal R., Karhu E., Wang J. S. (2019). Cell communication by tunneling nanotubes: implications in disease and therapeutic applications. *Journal of Cellular Physiology*.

[53] Wang J., Liu X., Qiu Y. (2018). Cell adhesion-mediated mitochondria transfer contributes to mesenchymal stem cell-induced chemoresistance on T cell acute lymphoblastic leukemia cells. *Journal of Hematology & Oncology*.

[54] Sáenz-de-Santa-María I., Bernardo-Castiñeira C., Enciso E. (2017). Control of long-distance cell-to-cell communication and autophagosome transfer in squamous cell carcinoma via tunneling nanotubes. *Oncotarget*.

[55] Whitehead J., Zhang J., Harvestine J. N., Kothambawala A., Liu G. Y., Leach J. K. (2020). Tunneling nanotubes mediate the expression of senescence markers in mesenchymal stem/stromal cell spheroids. *Stem Cells*.

