# Supplementary material for: Investigating Tunneling Nanotubes in Cancer Cells: Guidelines for Structural and Functional Studies through Cell Imaging
Source: Biomed Res Int. 2020 Apr 13;2020:2701345. doi: 10.1155/2020/2701345 (PMC7174938; doi:10.1155/2020/2701345)
Supplement: Supplementary 1 — Supplementary Figure 1: impact of cell density on TNT categories in PC12 cells. Supplementary Figure 2: adapted transmitted light microscopies for TNT observation. Supplementary Figure 3: formation of TNT1 in PC12 cells. Supplementary Figure 4: TNT1 and TNT2 imaging in living cancer cells through confocal microscopy. Supplementary Figure 5: confocal image processing and analysis for the determination of TNT features. Supplementary Figure 6: localization of actin and tubulin in TNT1 and TNT2 by double-labeling experiments and confocal microscopy. Supplementary Figure7: actin picks at the surface of TNT1 in cancer cells. Supplementary Figure 8: localization of cofilin in TNTs connecting HBEC-3 cells. Supplementary Figure 9: localization of vimentin in TNT1 connecting HBEC-3 cells. Supplementary Figure 10: localization of actin and tubulin in living PC12 cells through SiR tubulin probe. Supplementary Figure 11: improvement of lateral resolution through advanced light microscopy. [file 2701345.f1.pdf]

A **B**

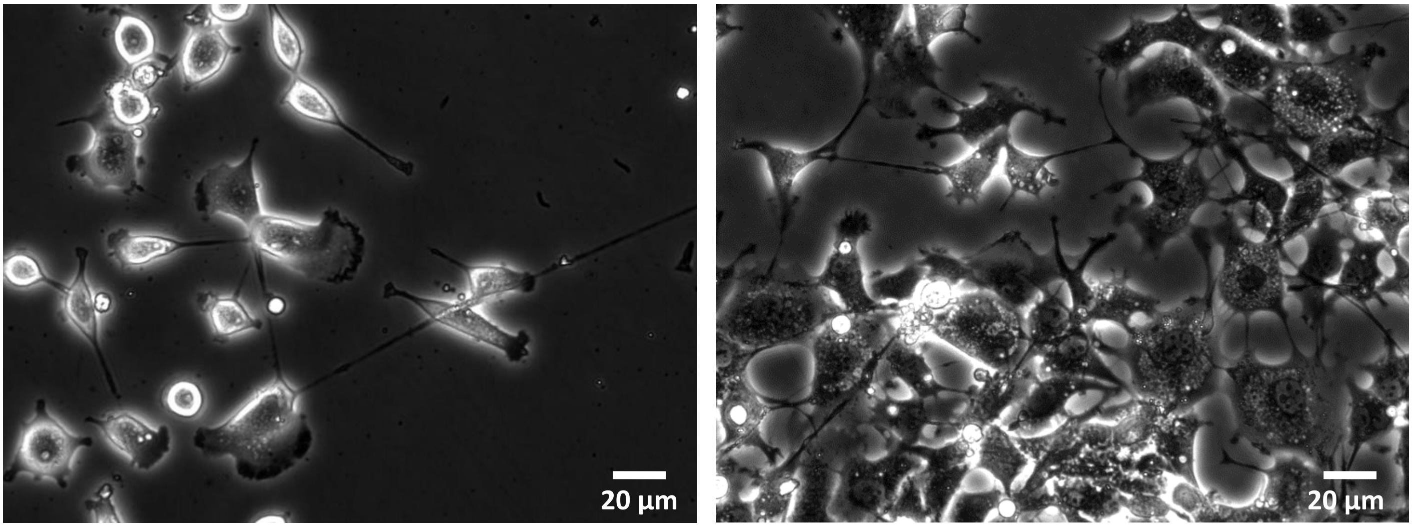

Supplementary Figure 1: Impact of cell density on TNT categories in PC12 cells. PC12 cells were cultured on plastic surface and images were acquired through phase contrast microscopy (DMI 6000 videomicroscopy, Leica Microsystems). Due to empty space on culture surface and cell proximity, a majority of TNT1 is observed one day after plating (**A**) when cell density was low and a higher number of TNT2 at later times when cell density increased (**B**).

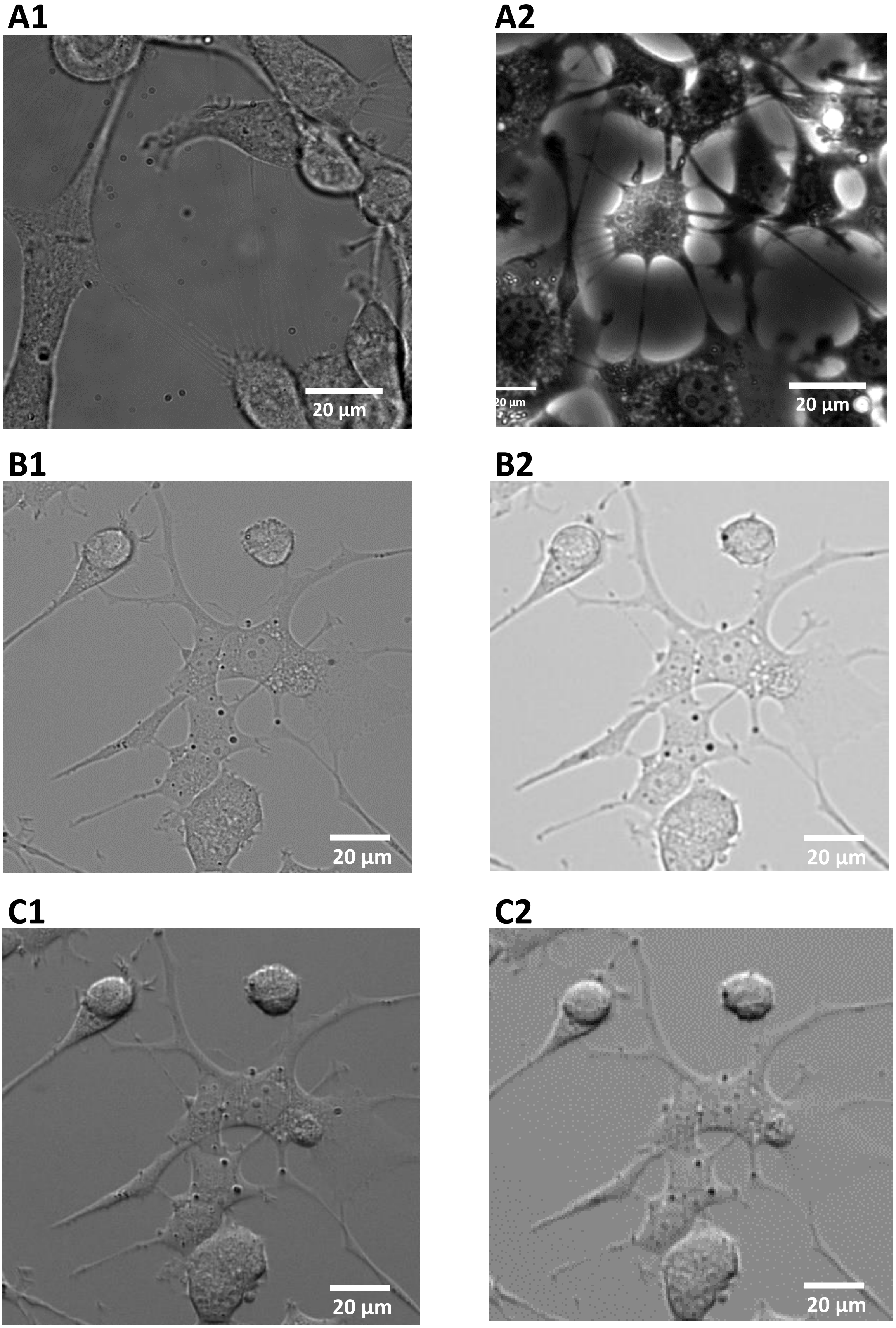

Supplementary Figure 2: Adapted transmitted light microscopies for TNTs observation. PC12 cells were cultured on glass surface and images were acquired through phase contrast (**A1, 63x**; **A2 20x+zoom 3, Leica videomicroscope**), bright-field (**B1, 40x**; **B2, 10X+zoom 4, Celldiscoverer 7, Zeiss**) or adaptative phase gradient contrast (**C1, 40x; C2, 10X+zoom 4, Celldiscoverer 7, Zeiss**) microscopy. Efficient contrast depends on objective type and phase devices.

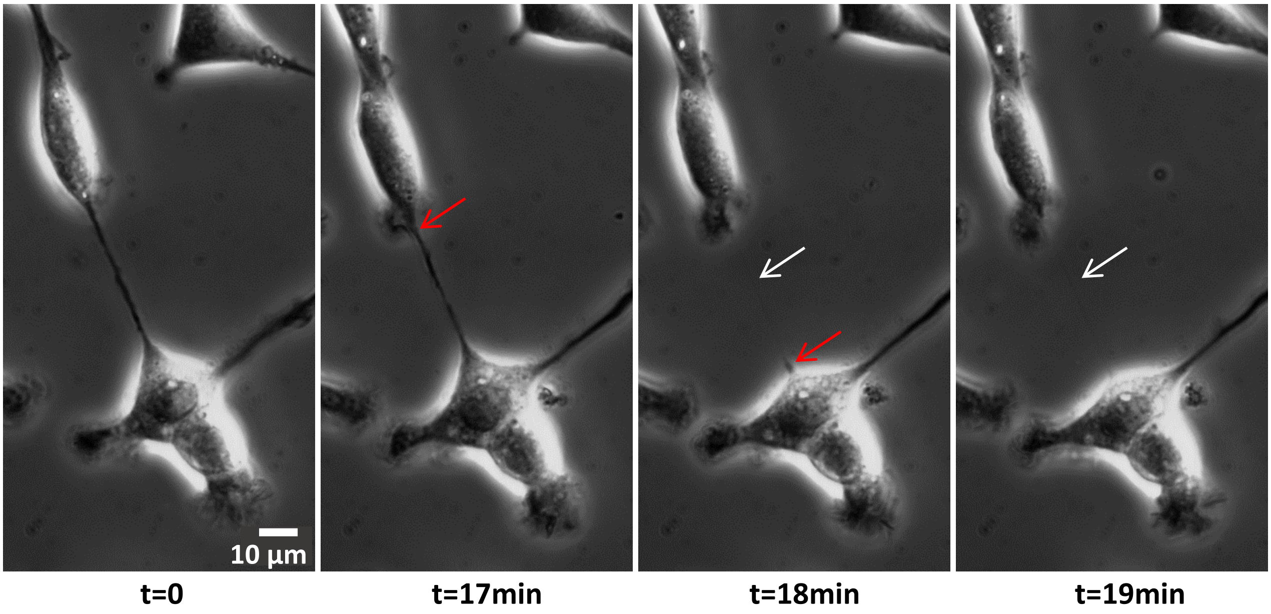

Supplementary Figure 3: Formation of TNT1 in PC12 cells. Migration of bulging portion along protrusion and formation of definitive TNT1 observed through transmitted light (phase contrast) videomicroscopy (dry objective x20). Red arrow indicates the bud. White arrow indicates the definitive TNT1. Scale bar, 20 µm.

A

B

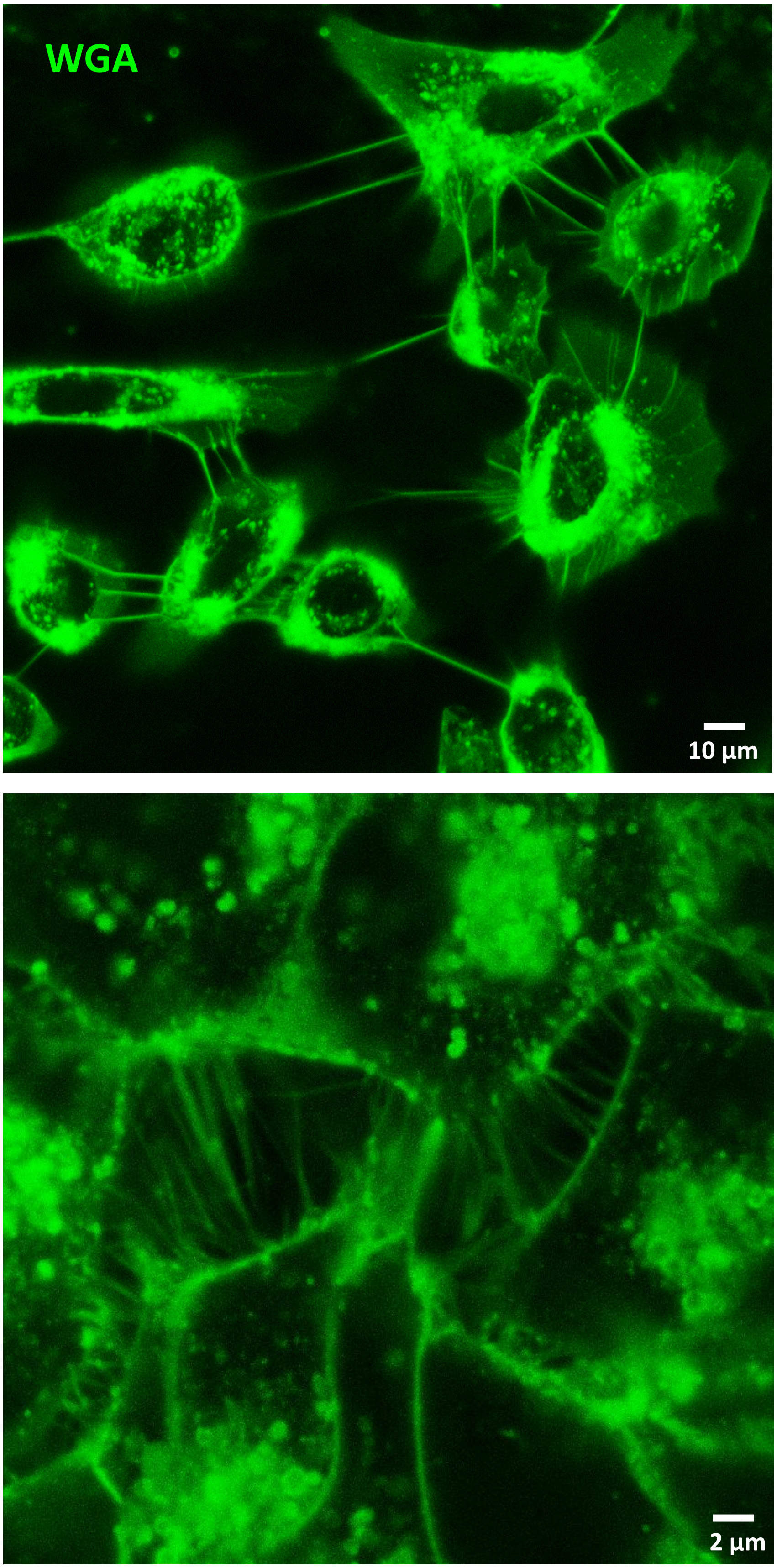

Supplementary Figure 4: TNT1 and TNT2 imaging in living cancer cells through confocal microscopy. Plasma membrane of living HBEC-3 (**A**) and PC12 (**B**) cells were labeled with Alexa-coupled wheat germ agglutinin (WGA) and Z-stack acquisitions (x,y,z) were performed through confocal microscopy (resonnant scanner, TCS SP5 X, Leica Microsystems).

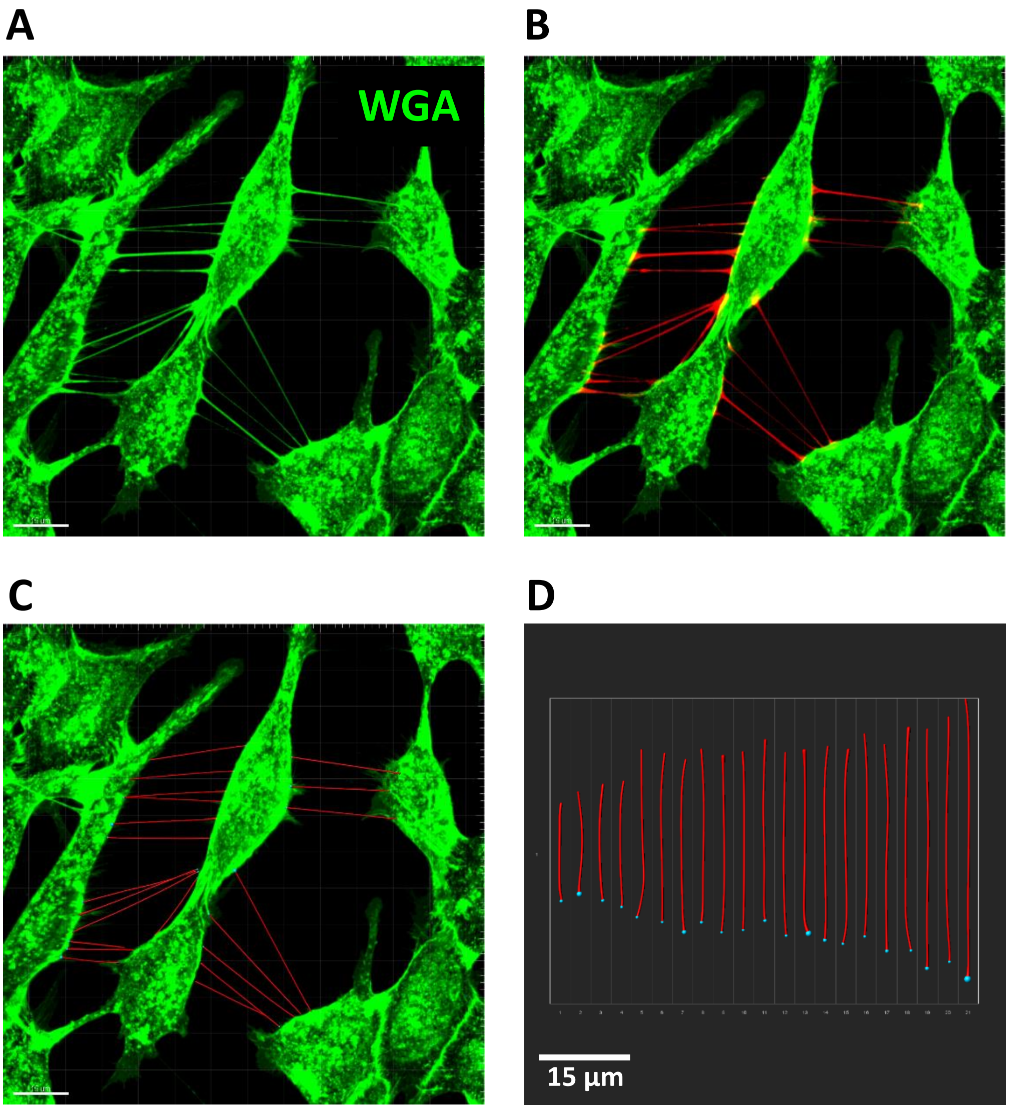

Supplementary Figure 5: Confocal image processing and analysis for determination of TNTs features. Top view in Imaris software (v 9.0.2, Bitplane) of deconvoluted confocal z-stack images illustrating Alexa 488-WGA labeled TNT1s within a network of living PC12 cells (**A**). Manual isolation and coloring (red) TNT1 through Imaris software (**B**). Semi-automatic TNT1 modeling (red tubes) between PC12 cells through Imaris (**C**). Automatic analysis of TNT number and length after modeling (**D**). Each red tube/TNT can be selected and consequently localized in figure C. Scale bar, 15 µm.

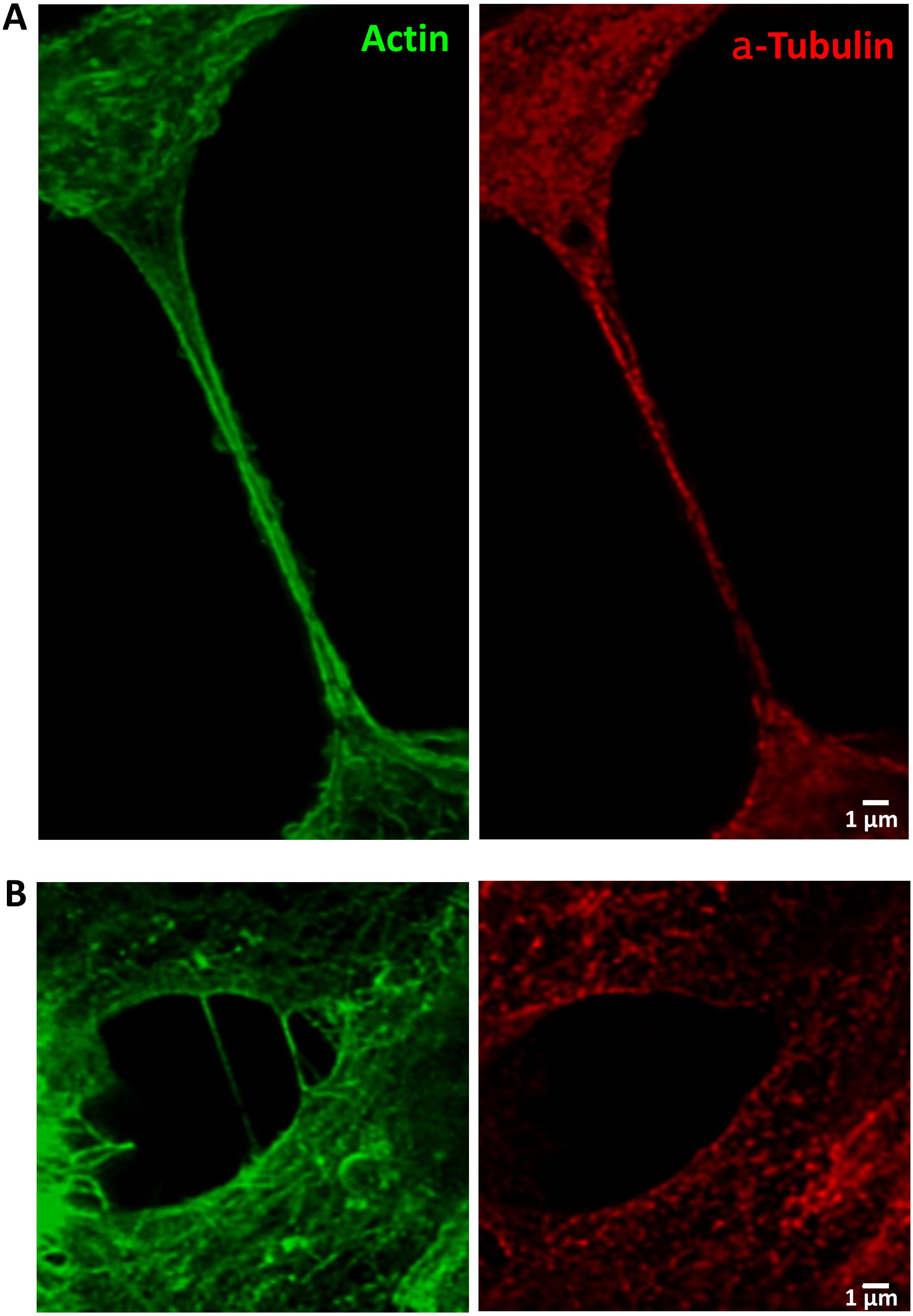

Supplementary Figure 6: Localization of actin and tubulin in TNT1 and TNT2 by double-labeling experiments and confocal microscopy. Actin (green) and tubulin (red) were detected from trumpet-shaped origins to the tip of TNT1 (**A**). In contrast to actin, tubulin was virtually absent from TNT2 (**B**). Scale bar, 1 µm.

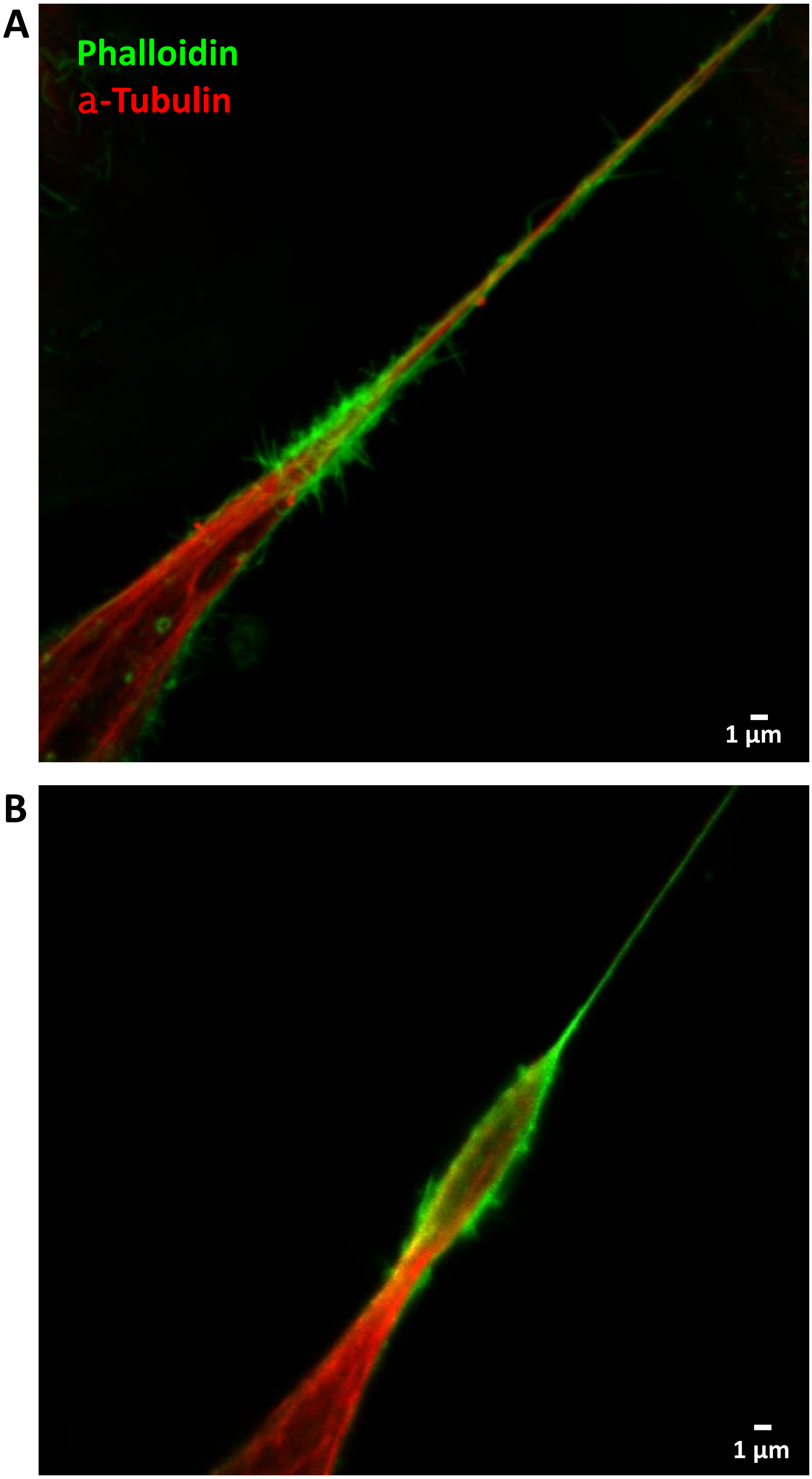

Supplementary Figure7: Actin-picks at the surface of TNT1 in cancer cells. H28 and HBEC-3 cells were fixed with PFA 4% and actin labeling was performed with Alexa-488 phalloidin and tubulin staining through immunodetection (Alexa-594). Image acquisition was obtained through confocal microscopy (TCS SP5 X, Leica Microsystems). The triangle origin of TNT1 in H28 cells (**A**) and the bulging portion of TNT1 closed to donor HBEC-3 cell body (**B**) were lined with short actin-picks. Scale bar, 1 µm.

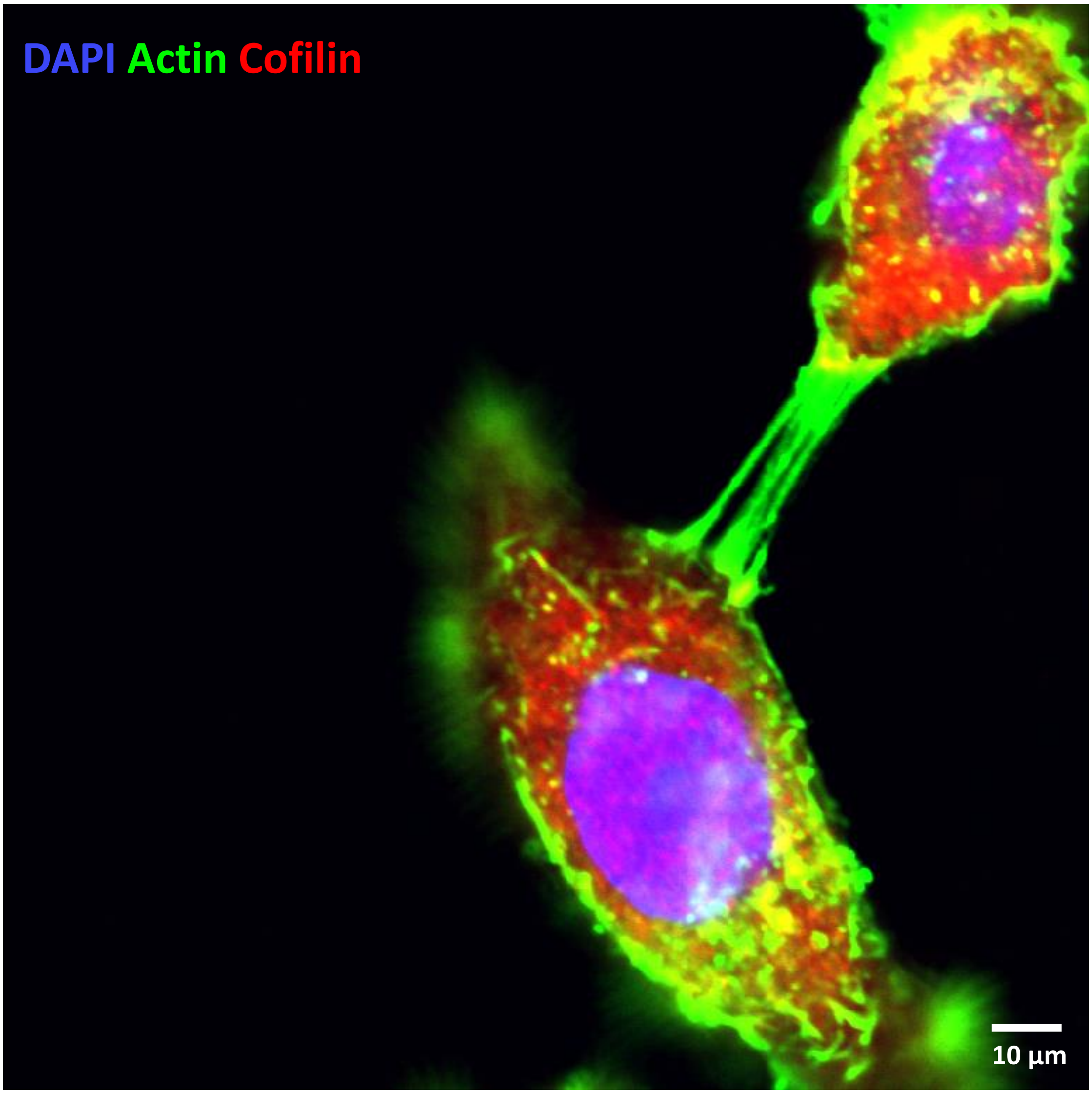

Supplementary Figure 8: Localization of cofilin in TNTs connecting HBEC-3 cells. HBEC-3 cells were fixed with PFA 4% then actin (green) or cofilin (red) was stained through immunodetection (Alexa-488 and Alexa-546 respectively). Nuclei were labeled with DAPI (blue). Image acquisition was captured with high-throughput confocal microscopy (FluoView FV1000, Olympus™). Scale bar, 10 µm.

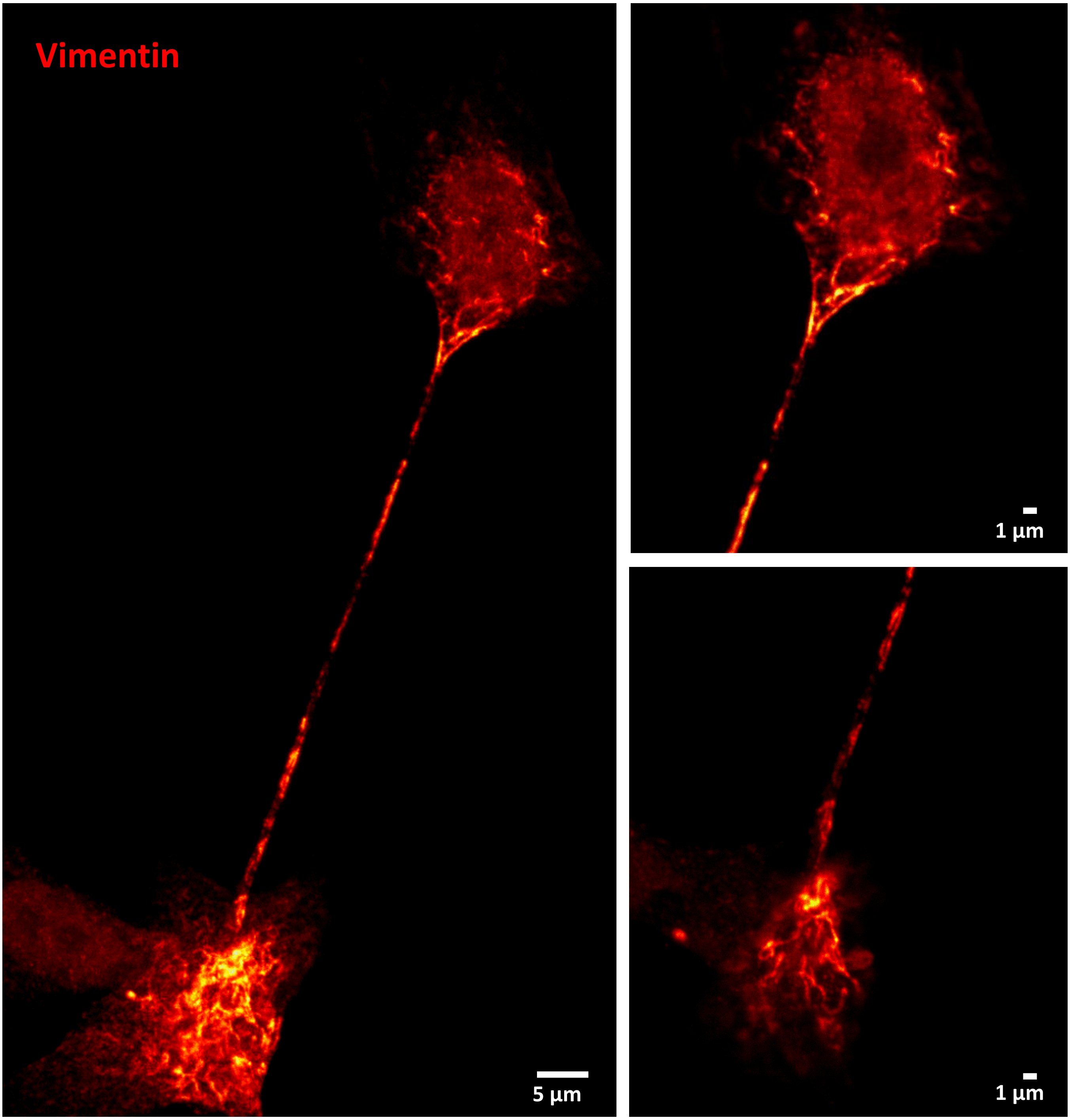

Supplementary Figure 9: Localization of vimentin in TNT1 connecting HBEC-3 cells. HBEC-3 cells were fixed with PFA 4% and vimentin staining through immunodetection (Alexa-488). Image acquisitions were obtained through confocal microscopy (TCS SP5 X, Leica Microsystems). Vimentin is detected in the donor cell, all along TNT1 and in the tip connecting the acceptor cells.

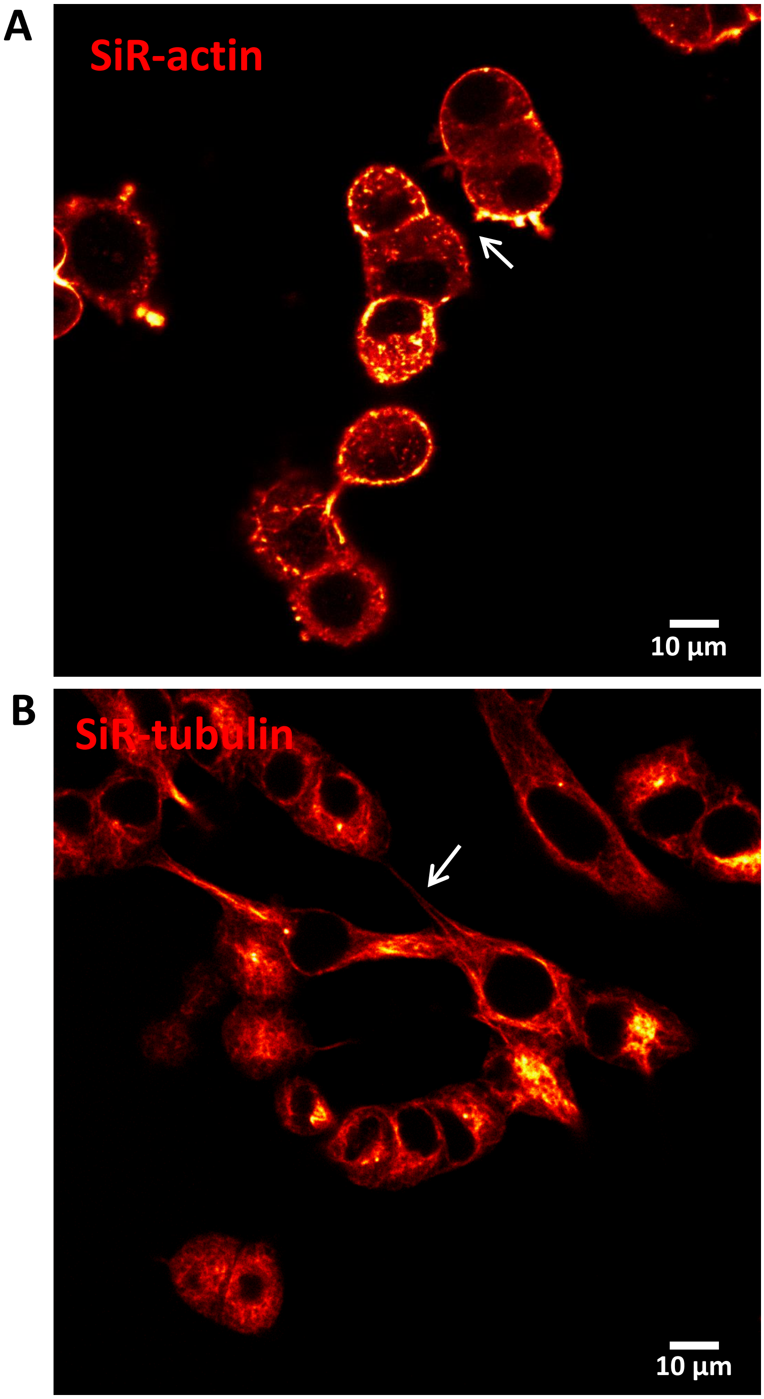

Supplementary Figure 10: Localization of actin and tubulin in living PC12 cells through SiR tubulin probe. Living PC12 cells were incubated with SiR actin (**A**) or SiR tubulin (**B**) at least 10 min and observed directly through confocal microscopy (TCS SP5 X, Leica Microsystems) equipped with an inverted stand. White arrows indicate labeled TNTs. Scale bar, 10 µm.

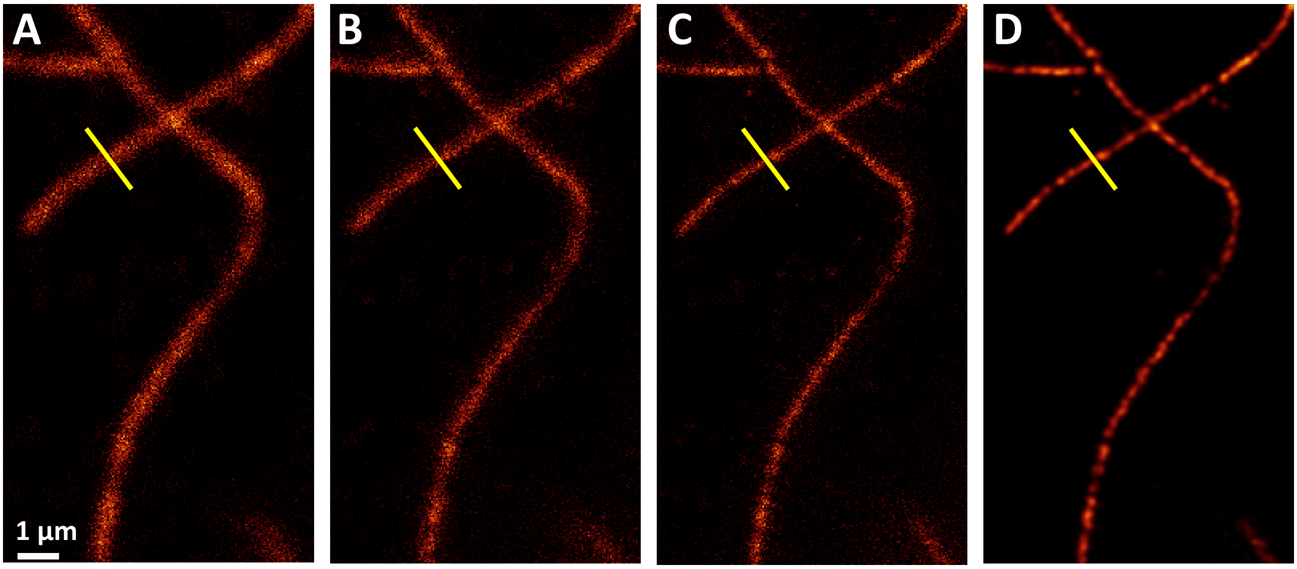

	Methods	µtubule diameter
A	Confocal	225 nm
B	CW STED	125 nm
C	Time-gated CW STED	93 nm
D	Deconvolved time-gated CW STED	76 nm

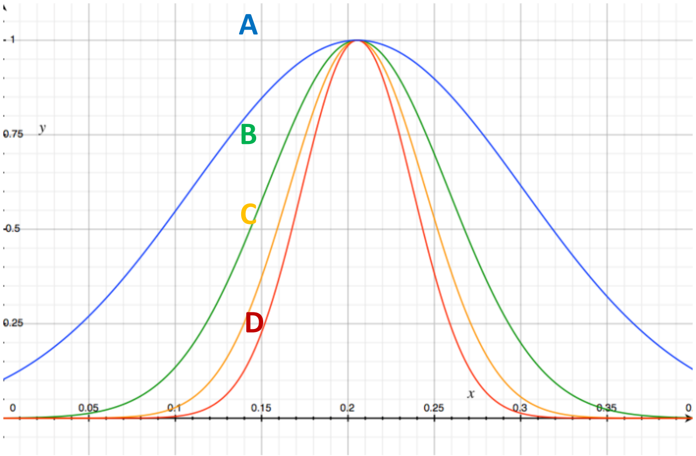

Supplementary Figure 11: Improvement of lateral resolution through advanced light microscopy. Evaluation of microtubule diameter in fixed PC12 cell with different strategies of cell imaging. (**A**) Confocal microscopy. (**B**) CW STED nanoscopy. (**C**) time-gated CW STED nanoscopy. (**D**) Deconvoluted time-gated CW STED nanoscopy. Graphic representation of the increase in lateral resolution obtained through the different approaches. The intensity profile was measured along the yellow segment and adjusted by using a Gaussian fit (Grapher, *i*OS). Tubulin was detected by using an indirect immunocytochemical staining with a second antibody conjugated to Alexa 488. Scale bar, 1 µm.
